# Small-Angle X-ray Scattering (SAXS) Measurements of APOBEC3G Provide Structural Basis for Binding of Single-Stranded DNA and Processivity

**DOI:** 10.3390/v14091974

**Published:** 2022-09-06

**Authors:** Fareeda M. Barzak, Timothy M. Ryan, Nazanin Mohammadzadeh, Stefan Harjes, Maksim V. Kvach, Harikrishnan M. Kurup, Kurt L. Krause, Linda Chelico, Vyacheslav V. Filichev, Elena Harjes, Geoffrey B. Jameson

**Affiliations:** 1 School of Natural Sciences, Massey University, Private Bag 11 222, Palmerston North 4442, New Zealand; 2 SAXS/WAXS, Australian Synchrotron/ANSTO, 800 Blackburn Road, Clayton, VIC 3168, Australia; 3 Department of Biochemistry, Microbiology and Immunology, University of Saskatchewan, Saskatoon, SK S7N 5E5, Canada; 4 Department of Biochemistry, University of Otago, P.O. Box 56, Dunedin 9054, New Zealand; 5 Maurice Wilkins Centre, University of Auckland, Auckland 1142, New Zealand

**Keywords:** APOBEC3, APOBEC3G, SAXS, protein–DNA complex

## Abstract

APOBEC3 enzymes are polynucleotide deaminases, converting cytosine to uracil on single-stranded DNA (ssDNA) and RNA as part of the innate immune response against viruses and retrotransposons. APOBEC3G is a two-domain protein that restricts HIV. Although X-ray single-crystal structures of individual catalytic domains of APOBEC3G with ssDNA as well as full-length APOBEC3G have been solved recently, there is little structural information available about ssDNA interaction with the full-length APOBEC3G or any other two-domain APOBEC3. Here, we investigated the solution-state structures of full-length APOBEC3G with and without a 40-mer modified ssDNA by small-angle X-ray scattering (SAXS), using size-exclusion chromatography (SEC) immediately prior to irradiation to effect partial separation of multi-component mixtures. To prevent cytosine deamination, the target 2′-deoxycytidine embedded in 40-mer ssDNA was replaced by 2′-deoxyzebularine, which is known to inhibit APOBEC3A, APOBEC3B and APOBEC3G when incorporated into short ssDNA oligomers. Full-length APOBEC3G without ssDNA comprised multiple multimeric species, of which tetramer was the most scattering species. The structure of the tetramer was elucidated. Dimeric interfaces significantly occlude the DNA-binding interface, whereas the tetrameric interface does not. This explains why dimers completely disappeared, and monomeric protein species became dominant, when ssDNA was added. Data analysis of the monomeric species revealed a full-length APOBEC3G–ssDNA complex that gives insight into the observed “jumping” behavior revealed in studies of enzyme processivity. This solution-state SAXS study provides the first structural model of ssDNA binding both domains of APOBEC3G and provides data to guide further structural and enzymatic work on APOBEC3–ssDNA complexes.

## 1. Introduction

APOBEC3 (A3) enzymes deaminate cytosine to uracil on single-stranded DNA [1] (ssDNA) or RNA [2]. There are seven family members in humans that act as part of the innate immune response against viruses and retrotransposons where ssDNA deamination is the primary substrate [1,3,4,5]. The presence of 2′-deoxyuridine in ssDNA templates results in the addition of thymidine during DNA replication, causing C→T mutations. Due to their promutagenic nature, this enzyme family is a double-edged sword. For example, some members of this family, such as A3A and A3B, can become dysregulated and induce mutation of host DNA and are linked to cancer development, cancer evolution and drug resistance [6,7]. However, the seven human A3 enzymes (A3A-A3G, except for E) are primarily known for their ability to restrict the replication of HIV. A3G was the first member discovered from this family and is a potent anti-HIV restriction factor [8]. Its significance is so great that the HIV protein, Vif, among other functions, mimics the substrate receptor of a Cullin 5 ubiquitin ligase to target A3G, and other A3s, for degradation [9,10,11]. A3G potently restricts Vif-deficient HIV [12] and can also induce mutations in Vif + HIV, but the contributions of the mutations to viral inactivation or virus escape and drug resistance are not clear [13,14,15,16]. A3G and other A3 enzymes restrict their relevant pathogens using both a deaminase-dependent and a deaminase-independent mechanism [8,17]. The deaminase-dependent function is based on the deamination of cytosine, and the deaminase-independent function is based on physically inhibiting reverse transcriptase activity by oligomerizing on ssDNA or RNA or, additionally for A3G, by a direct interaction with the reverse transcriptase itself [18,19,20,21]. Complicating in vitro studies, A3G is usually purified as a high-order multimer with low activity, which dissociates after RNAse A treatment to a mixture of lower-order oligomeric states with higher activity [22]. The oligomerization of A3G and other A3 proteins was recently reviewed by Chen [20].

A3G contains two zinc(II)-binding deaminase domains, of which only the C-terminal domain (CTD) is catalytically active and responsible for deamination of cytosine. On the other hand, the N-terminal domain (NTD) is in charge of RNA binding, virion encapsulation [23], processivity on ssDNA [24] and interaction with Vif [24,25]. For long DNA fragments (>60-mer), A3G shows a processive behavior [22,24,25], where it scans its substrate for the deamination motif (-CCC-), preferentially located near the 5′ end of DNA, by a sliding and jumping [22] mechanism, as well as by inter-segmental transfer [26]. A remarkable, approximately 30-mer “dead zone” for nucleotides 3′ of the CCC deamination motif had been revealed earlier [27]. A3G is also shown by Förster resonance energy transfer (FRET) to hover over the deamination hotspot [28]. The CTD of A3G by itself is weakly active, whereas the activity and processivity are greatly enhanced by the presence of the catalytically inactive but structurally homologous NTD in full-length A3G [23,27,29,30,31].

Recently, the crystal structures have been published of two-domain full-length A3G, denoted A3G_fl_. One set of structures is from human A3G_fl_ (PDB: 6WMA, 6WMB, 6WMC) [32]; the other set of structures is from rhesus monkey (PDB: 6P40, 6P3Z, 6P3X, 6P3Y) [33]. Both sets of A3G_fl_ structures are heavily modified for stability and solubility, and only one structure has both Zn^2+^ binding sites occupied (6P40). Individual domains of A3G, both with [34] and without ssDNA [35,36,37,38,39,40], have also been structurally characterized. So far, however, there have not been any experimentally based models and structures of full-length two-domain *active* enzymes with ssDNA bound.

The effect of ssDNA on A3G oligomerization has been identified biochemically and under single-molecule conditions to be dynamic [19,41,42]. Analytical centrifugation has shown that apo A3G is mostly dimeric with smaller number of monomers and tetramers present, depending on concentration [43], but the structure of tetramers is not known. Extensive prior research has been conducted on the interaction of A3G with RNA and how this affects DNA interaction, but this is not the focus of this study. Here, we apply small-angle X-ray scattering (SAXS) with co-flow [44] combined with size-exclusion chromatography (SEC-co-flow-SAXS) to study full-length A3G in the presence and absence of ssDNA. In addition to model-free biophysical parameters, such as the radius of gyration (*R*g), maximum dimension and molecular weight of the protein that are derived from the SAXS scattering data [45], we also derive from the data a model for the primary species in solution and present a model on how A3G interacts with ssDNA. The data support a model in which A3G_fl_ is composed of multiple oligomeric states in solution and bound to ssDNA [19,41,42,43]. However, binding to ssDNA converts the free multimeric apo-A3G_fl_ to a state in which there are relatively more monomeric species bound to ssDNA. There is some discussion in the field with respect to which oligomeric state is more active [19,42]. Our data confirm that ssDNA is an active player in altering the oligomeric state of A3G_fl_ [19,41,42] and show that ssDNA initiates the formation of the catalytically active monomer [27].

## 2. Materials and Methods

### 2.1. Protein Preparation for SAXS

Glutathione S-transferase (GST) fused to A3G_fl_, GST-A3G_fl_, was produced using a recombinant baculovirus and *Sf*9 cell system as described previously [22,27]. *Sf*9 cells were infected with recombinant A3G_fl_-expressing virus at a multiplicity of infection of 1 and harvested after 72 h. Cells were lysed as described previously in the presence of 100 μg mL−1 of RNase A. Cleared lysates were then incubated with glutathione-Sepharose resin (GE Healthcare, Chicago, IL, United States) and subjected to a series of salt washes (0.25–1 M NaCl) before on-column cleavage of the GST tag with 40 units of thrombin (Calbiochem, San Diego, CA, United States) for 16 h at 21 °C. Cleaved wild-type A3G_fl_ fractions were further purified by size-exclusion chromatography (SEC) using a G200 Increase (GE Healthcare) column with the following running buffer: 50 mM HEPES, pH 7.2, 200 mM NaCl, 10% glycerol, 1 mM DTT and 150 mM L-arginine HCl. Fractions were concentrated using Amicon Ultra Centrifugal Filter units and stored at −80 °C.

### 2.2. Protein Purification for Activity Assay

A3G_fl_ was produced as previously described [46,47]. Briefly, HEK293-6E cells were grown planktonically, following transient transfection, as described previously [47], using a pTT5 protein expression plasmid coding for GST-tagged A3G_fl_. Soluble A3G_fl_ for activity/inhibition assays was purified from lysed HEK cells using a 5 mL GSTtrap FF column (Cytiva Marlborough, MA, United States). After removal of the GST tag with PreScission protease, the sample was then further purified using a Superose 12 column.

### 2.3. DNA Synthesis

The substrate 5′-ATTCCCAATT and inhibitors 5′-ATTCCdZAATT (abbreviated CCdZ-oligo) and 5′- ATTCC5FdZAATT (5FdZ is 5-fluoro-2′-deoxyzebularine; abbreviated CC5FdZ-oligo) were prepared as previously described [48]. Higher oligomeric 40-mer DNAs, 5′-ATTCCdZAATT-T30, 5′-ATTCC5FdZAATT-T30, 5′-T30-ATTCCdZAATT and 5′-T30-ATTCC5FdZAATT containing dZ and 5FdZ were similarly synthesized (and, respectively, abbreviated CCdZ-T30-oligo, CC5FdZ-T30-oligo, T30-CCdZ-oligo and T30-CC5FdZ-oligo).

### 2.4. NMR Inhibition Assay

The NMR inhibition assay was performed at 298 K, as previously described [48], using a 700 MHz Bruker NMR spectrometer equipped with a 1.7 mm cryoprobe. The initial speed of deamination of 500 μM 5′-ATTCCCAATT (target dC underlined) by ~450 nM A3G_fl_ in the absence or presence of 50 μM CCdZ- or CC5FdZ-containing oligos (10-mer and 40-mer) was measured. NMR experiments were conducted in a pH 6.0 kinetic buffer (100 mM NaCl, 50 mM sodium phosphate buffer, 10 % D_2_O, 1 mM citrate, 50 μM sodium trimethylsilylpropanesulfonate (DSS)).

### 2.5. SAXS Data Acquisition and Analysis

A3G_fl_ was initially assessed at 2.5 mg/mL (slight aggregates formed), and SEC caused a dilution, so a new concentration of approximately 1.3 mg/mL was calculated based on the absorbance at 280 nm. SAXS measurements were conducted at the Australian Synchrotron on the SAXS/WAXS beamline equipped with a Pilatus-2 1M detector as described [45]. Samples were run at 25 °C through a SEC column (Superdex™ 200 Increase 5/150 GL, GE Healthcare) at a flow rate of 0.2 mL/min in A3G_fl_ SAXS buffer (50 mM phosphate, pH 6.0, 200 mM NaCl, 2 mM β-mercaptoethanol (β-ME), 5% glycerol, 200 µM Na_2_-EDTA).

Glucose isomerase data were collected as control to confirm that that SAXS setup worked correctly, and water was run to provide calibration for the absolute intensity of scattering. SAXS measurements were obtained at 25 °C using a camera length of 1.6 m, and frames were taken at one-second intervals. SVD and EFA modules (SVD/EFA BioXTAS RAW [49]) were used to elucidate the number and boundaries of the scattering components for the ligand-free A3G_fl_ and A3G_fl_ with a 40-mer ssDNA as described in Barzak et al. [45]. The only difference is that the Gaussian analysis was performed afterward to discriminate between overlapping species using US-SOMO [50,51,52], as described in great detail in the Supplementary Information of Brookes et al. [50]. The Gaussians that produced good scattering data were analyzed using PRIMUSQT (ATSAS 2.8.3 suite) to identify the species present as described previously in Barzak et al. [45]. In addition, SAXS data were collected on the 40-mer ssDNA by itself. All deconvolution methods have limitations, which we largely mitigated by using two independent approaches to deconvolute the data SVD/EFA and Gaussian analysis, where SVD ignores the time dimension of the data set, and Gaussian decomposition relies entirely on the time profiles of the scattered intensities [51]. For further discussion on the limitations, see Brooks et al. [50,51].

## 3. Results

### 3.1. SEC-SAXS Analysis of Apo A3G_fl_: Deconvolution of Scattering Data

**Figure 1:**
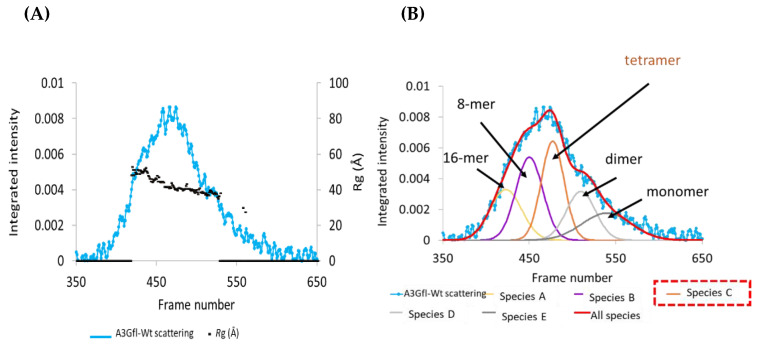
Deconvolution of the SEC-SAXS data of A3G_fl_. (**A**) SAXS profile superimposed with *R*g trace values. (**B**) Gaussian decomposition analysis using US-SOMO. Experiments were conducted at 25 °C using 2.5 mg/mL A3G_fl_ in A3G_fl_ SAXS buffer (50 mM phosphate, pH 6.0, 200 mM NaCl, 2 mM β-ME, 5% glycerol, 200 µM Na_2_-EDTA). See text below and Figure S3 (Supplementary Materials) for evidence supporting Gaussian decomposition and assignment of multimers and monomer.

The initial characterization of the catalytically active full-length wild-type ligand-free A3G protein using both the UV elution profile and the SAXS profile indicated that the sample had multiple components that were incompletely resolved by size-exclusion chromatography (SEC) (Figure 1A,B and Figure S1A,B). Superimposing the radius of gyration, the *R*g, trace values over the elution and SAXS profiles revealed a large variability in these values from ~30 to 50 Å across this peak, indicating that multiple scattering species were present in the sample (Figure 1A and Figure S1A,B). Therefore, before further analysis, the species were separated by deconvolution as detailed elsewhere [45] (see also Supplementary Materials and Materials and Methods).

The derived scattering curves had a low signal–noise ratio because of deconvolution and the resultant low concentration of each species. Although the data were fitted with five species, only Species-B and -C (magenta and orange) produced acceptable scattering curves and showed higher intensities than the other species, indicating their greater contribution to the overall scattering pattern of the sample. Therefore, these two curves were further analyzed to identify the corresponding oligomeric states.

### 3.2. SAXS Analysis of Apo A3G_fl_: Analysis of the Scattering Curves

The initial analysis of the derived scattering curves revealed that the A3G_fl_ scattering species were homogeneous, illustrated by the plateau at low *q* in the double log graph (see Figure S2B in Supplementary Materials, see also text below). From the *P*(*r*) plot [53], A3G_fl_-Species-B was estimated to have an *R*g ~ 60 Å and *D*max ~ 160 Å. However, as the scattering data of this species had a low signal-to-noise ratio, the accurate derivation of the structural parameters for this species was difficult (see Figure S2A,B in Supplementary Materials).

The comparison of the Kratky plots [53] of the derived scattering species showed that the position of the peak maximum of the A3G_fl_-Species-B (at *q* ~ 0.025 Å^−1^) was half that of the A3G_fl_-Species-C (*q* ~ 0.05 Å^−1^) (see Figure S2C in Supplementary Materials), indicating that the A3G_fl_-Species-B was about double the size of the A3G_fl_-Species-C. The higher value here indicates the smaller size, as scattering vector *q* is related to the inverse size. The same trend was also observed in the elution pattern, as the bigger particles elute off the SEC column earlier than smaller ones; A3G_fl_-Species-B elutes before A3G_fl_-Species-C (Supplementary Figure S1D). Finally, notwithstanding the similar intensity of X-ray scattering from the two species, this intensity is biased by the sixth-power relationship between the particle size and the scattering of electromagnetic radiation. Thus, A3G_fl_-Species-C was present in higher concentrations than A3G-Species-B, leading to a better signal-to-noise ratio (Figure S2).

We then focused on the more prominent A3G_fl_-Species-C, for which we found a satisfactory Guinier plot indicating a monodisperse sample (see Figure S2B insert and Figure S2E in Supplementary Materials). Both the *R*g and *I*(0) derived from the Guinier and the *P*(*r*) plots were very similar, confirming the relative size of the species as listed in Table S1. The Kratky plot showed a nice bell-shaped curve with a peak maximum *q* ~ 0.05 Å^−1^; however, the plot at higher *q* did not completely return to the baseline, suggesting that the structure may contain flexible regions (see Figure S2C in Supplementary Materials). The *R*g-normalized Kratky plot [54] shows that the peak maximum sits at the position indicative of a well-folded globular protein ((√ 3, 1.104); Figure S2F). Noticeably, the estimated values of *R*g and *D*max of this A3G-Species-C (*R*g ~ 42 Å, *D*max ~ 146 Å, Table S1) were found to be similar to a previously reported elongated dimer for A3G_fl_ derived from SAXS data [55]. However, our molecular weight was estimated to be ~208 kDa (derived from the Porod volume, not from *I*(0) [56], as each species is overlapped, as discussed above), which was double the molecular weight previously reported, using non-deconvoluted data, for an A3G_fl_ elongated dimer of ~100 kDa [55]. This suggested that our A3G_fl_-Species-C is not a dimer but a tetramer under our conditions, in which four two-domain A3G_fl_ molecules oligomerize together. The DAMMIF-derived ab initio envelope calculated from the SAXS data for the tetramer species, and the structural model derived, will be discussed below in context with the complex between the tetrameric species and ssDNA (see also Supplementary Materials, Figure S4).

To identify the oligomeric states of the other deconvoluted species (and as an orthogonal by-product to validate the deconvolution into potentially five multimeric species of A3G_fl_), we plotted the logarithm of the oligomerization number as a proxy for molecular weight (log1 for a monomer, log2 for a dimer, etc.) as a function of the frame number at the maximum of scattering for each of the species deconvoluted (frame number is proportional to the elution time in SEC (Supplementary Figure S3)). As we already identified Species-C as a tetramer, the smaller species were assigned to a dimer and monomer; the bigger are likely to be 8-mer and 16-mer, as the simplest explanation for oligomers would be the multiples and fractions of the tetramer. The resulting plot, effectively an SEC calibration plot, should be linear if our assumption of multimeric A3G_fl_ species is correct. Our plot clearly shows linear dependency, confirming our assignment of oligomerization states (Figure 1B), especially for the dimer and monomer species. As a logarithmic scale “flattens” the data, the assignment of 8-mer and 16-mer, while tentative, is, for lack of a better adverb, oligomerizationally sensible. Taken together, our SEC-SAXS experiments show that the ligand-free double-domain A3G_fl_ exists in multiple quaternary states in solution, and one of the two dominant scattering species, Species-C, is a tetramer. The other dominant species in scattering, Species-B, with a much larger *R*g, is a higher-order multimer (likely 8-mer). Our deconvolution data are roughly similar to the analytical ultracentrifugation data of Salter at al. [43], where dimers were prevalent, but tetramers and monomers were also present, and their amounts depended on the protein concentration. As SAXS is biased toward high molecular weights (the intensity is biased by the sixth-power relationship between the particle size and the scattering of electromagnetic radiation), the scattering is heavily dominated by larger molecules. Accordingly, the tetramer becomes the prevalent scattering species. We cannot completely exclude that a residual amount of RNA may be responsible for the higher-order multimers, but the prevalence of higher-order oligomers should be very low, as higher-order multimers dominate the scattering.

### 3.3. SAXS Model of A3G_fl_ in Complex with ssDNA

#### Selection of ssDNA for SAXS Studies

To prevent deamination of ssDNA during SAXS experiments on active A3G_fl_, we decided to use chemically modified DNA [57], that is, an inhibitor species. A similar approach was used by us to study the catalytically active C-terminal domain (CTD) of A3B, A3B-CTD, in complex with ssDNA, where dZ replaces the target dC [45]. A3G_fl_ has an intrinsic preference toward deamination of dC at the 3′-end of a CCC-motif (*K*m ~ 570 µM for A3G-CTD acting on the 10-mer substrate 5′-ATTCCCAATT, abbreviated CCC-oligo [57]). The incorporation of dZ or its 5-fluoro derivative (5FdZ) at the 3′-end of the CCC-motif on the 10-mer oligonucleotide (5′-ATTCCdZAATT, abbreviated CCdZ-oligo, and 5′-ATTCC5FdZAATT, abbreviated CC5FdZ-oligo) led to significant inhibition of A3G-CTD activity on the CCC-oligo by CCdZ-oligo [46,58] (and somewhat less so by CC5FdZ-oligo), as dZ and FdZ cannot be deaminated. dZ and FdZ form a tetrahedral intermediate in the active site of A3, as evidenced in the recent crystal structures of FdZ oligo with wild-type A3A [59] and dZ oligo with A3G-CTD [60]. The observed deamination was a result of the residual activity of A3G-CTD on the 10-mer CCC-substrate. The cytosines in our CCdZ- and CC5FdZ inhibitors are not deaminated by A3 enzymes because the *K*_i_ for dZ in CCdZ-containing oligos is much lower than the *K*_m_ for the remaining cytosines in these motifs [48,58].

**Figure 2:**
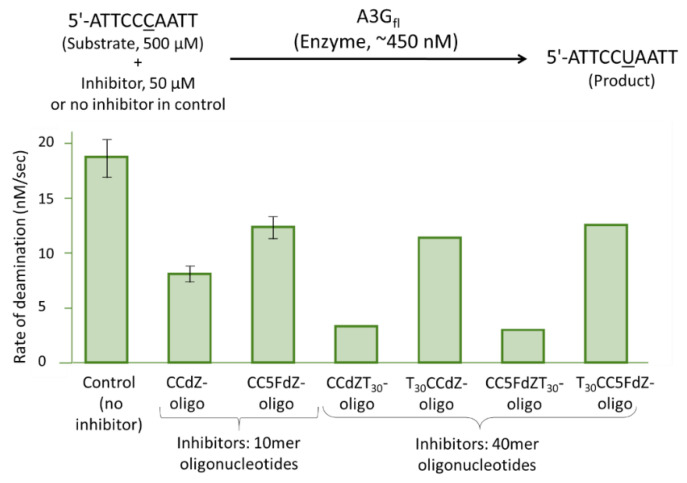
Qualitative screen of modified oligos on the inhibition of the A3G_fl_-catalyzed deamination of the preferred dC substrate 5′-ATTCCCAATT (CCC-oligo). Plot of the initial speed of deamination of 500 μM 5′-ATTCCCAATT (target dC underlined) by the A3G_fl_ in the absence or presence of 50 μM CCdZ- or CC5FdZ-containing 10- and 40-mer oligos. Experiments were performed using the ^1^H-NMR-based inhibition assay in pH 6.0 kinetic buffer (100 mM NaCl, 50 mM sodium phosphate, 10 % D_2_O, 1 mM citrate, 50 μM DSS) at 298 K. Experiments with 10-mer oligonucleotides were repeated multiple times, and the mean values were plotted with error bars reported as SD, whereas single experiments were performed on the 40-mer oligos. Full inhibitor sequences are given in Materials and Methods.

Noting that 20-mer oligonucleotides had been reported to have higher binding affinities to a single-domain A3A/A3B chimeric construct than shorter oligonucleotide sequences [48], we therefore thought a 40-mer oligonucleotide would better accommodate binding to double-domain A3G_fl_. A 40-mer oligonucleotide containing the sequence of 10-mer CCdZ-oligo or CC5FdZ-oligo preceded by a poly T30 tail at the 5′-end (T30-CCdZ-oligo and T30-CC5FdZ-oligo) decreased the deamination rates of the substrate CCC-oligo by A3G_fl_ by a similar factor to the control oligonucleotides CCdZ-oligo and CC5FdZ-oligo (Figure 2) [46]. However, the placement of the CCdZ- or CC5FdZ- motif near the 5′-end of the 40-mer ssDNA (CCdZ-T30-oligo and CC5FdZ-T30-oligo) led to a more pronounced inhibition of A3G_fl_, consistent with the reported polarity of A3G-induced deamination [41].

The 5FdZ-containing ssDNA did not further improve the inhibition of A3G_fl_ over dZ-containing ssDNA, neither by the 10-mer [46] nor by the 40-mer oligo (Figure 2). Therefore, to study the structure of the double-domain A3G_fl_ in complex with ssDNA using SAXS, the 40-mer CCdZ-T30-oligo was selected to ensure binding to the CTD in a productive conformation to cause inhibition of the catalytically active A3G_fl_.

### 3.4. SAXS Studies of CCdZ-T_30_-Oligo

**Figure 3:**
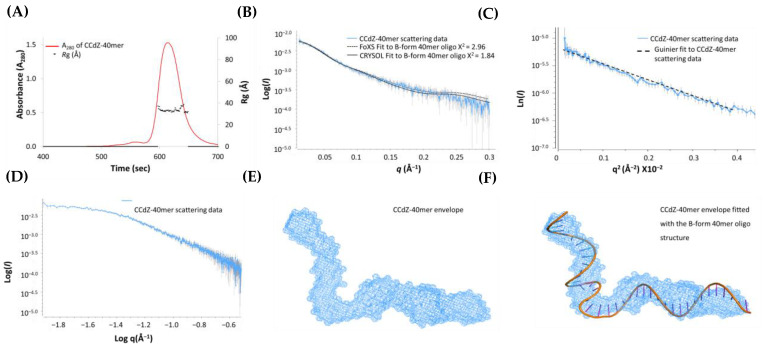
SEC-SAXS analysis of CCdZ-T30-oligo. (**A**) SEC elution profiles (red) of CCdZ-T30-oligo (further abbreviated as CCdZ-40 mer in the figure), with *R*g trace (black dots) superimposed. Only the data between the thickened black lines on the *x*-axis were retained for analysis. (**B**) Fits of a kinked B-DNA model to SAXS X-ray data (blue) for the CCdZ-T30-oligo using FoXS (dashed black line) and CRYSOL (solid black line). (**C**) Guinier plot of CCdZ-T30-oligo scattering data (blue) and its fit (dashed black line). (**D**) Double log plot of CCdZ-T30-oligo scattering data. (**E**) Averaged envelope model of CCdZ-T30-oligo assuming P1 symmetry using DAMMIF and refined with DAMAVER and DAMFILT (ATSAS 2.8.3 suite). (**F**) Envelope model (blue shape) superimposed with the B-form of DNA for CCdZ-T30-oligo (centipede cartoon model).

Initially, the CCdZ-T30-oligo was examined using SEC-co-flow-SAXS to understand its dynamic structure in solution and potentially to aid in modeling the A3G_fl_–ssDNA complex. The CCdZ-T30-oligo eluted off the SEC column as a single monodisperse species (Figure 3A), with a steady *R*g ~32 Å displayed across the peak in both the elution and SAXS profiles (Figure 3A and Figure S5A). The double log plot resulted in a plateau at low *q* values, indicating that the sample was homogeneous (see Figure 3D), which was further verified by a good fit with the linear regression in the Guinier plot [53] (Figure 3C).

The estimation of the *R*g and *I*(0) values from the Guinier slope agreed well with the values obtained by the independent *P*(*r*) method, further confirming the quality and relative size of the oligonucleotide listed in Table S3 (Supplementary Materials). From these parameters, the estimated MW ~12–13 kDa of the oligonucleotide was found to be comparable to the expected MW ~12 kDa. The Kratky profile [53] indicated that the oligonucleotide adopted a highly flexible extended conformation, as the scattering intensity at values of *q* > 0.1 Å^−1^ did not return to the baseline (see Figure S5C in Supplementary Materials). This was additionally supported by the *P*(*r*) plot [53], which was significantly skewed to the right in comparison to the standard symmetrical bell-shaped histogram for a compact quasi-spherical moiety [53] (see Figure S5D in Supplementary Materials).

As free DNA may have a reasonably long persistence length [61], we performed the reconstruction of the scattering profile using 3D envelope modeling. This reconstruction of the scattering profile (NSD ~ 0.875) demonstrated, remarkably, a well-defined shape for the oligonucleotide. The envelope model mimics a dumbbell, which is consistent with the shape of the *P*(*r*) curve (see Figure 3E and Figure S5D), as described in the literature [62]. This model illustrates that the oligonucleotide is single stranded and is made up of approximately four helical turns. The model described is likely a representation of a smeared conformational distribution, and further work using an ensemble approach with higher-quality data may provide better elucidation of the conformational space available to the oligo.

Based on the envelope model, the CCdZ-T_30_-oligo adopts, under our conditions in solution, an approximately standard B-form DNA conformation (Figure 3E), even though it is single stranded. Therefore, the averaged envelope model was superimposed with a B-form 40-mer ssDNA structure (designed using the *make na* server http://structure.usc.edu/make-na/server.html, accessed on 1 April 2019) and modeled with a kink. The 40-mer ssDNA structure gave a remarkably good visual fit with the SAXS-derived molecular envelope (Figure 3F). To validate this model, the observed CCdZ-T_30_-oligo SAXS scattering profile was compared with the back-calculated 1D scattering profiles of the designed B-form ssDNA. The scattering data showed a good visual fit with this model, especially in the *q* regions between 0.02 and 0.2 Å^−1^, as illustrated in Figure 3B and summarized in Table S4. Therefore, the in-solution SAXS-based model of the CCdZ-T_30_-oligo is comparable to the rigid B-DNA model structure.

### 3.5. SAXS Model of A3G_fl_ in the Presence of dZ-Containing ssDNA

**Figure 4:**
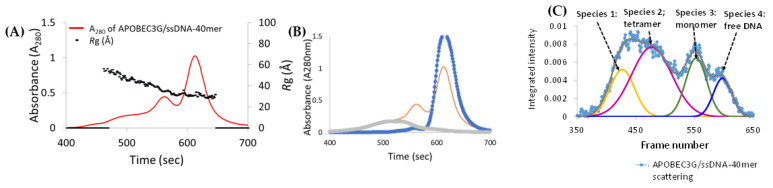
SEC-FLPC profile comparison of apo A3G_fl_ and A3G_fl_/ CCdZ-T_30_-oligo shows complex formation. (**A**) SEC-FPLC elution profile of A3G_fl_ with CCdZ-T_30_-oligo (red, abbreviated as ssDNA-40mer in the figure) at a 1 to 2 molar ratio, with *R*g trace values (black dots) superimposed. Only the data between the thickened black lines on the *x*-axis were retained for analysis. (**B**) Superposition of SEC-FPLC elution profiles of A3G_fl_ alone (gray), of CCdZ-T_30_-oligo (blue) and of A3G_fl_ with CCdZ-T_30_-oligo (abbreviated as ssDNA-40mer in the figure) at a 1 to 2 ratio (orange). (**C**) Gaussian decomposition analysis using US-SOMO. Experiment conducted using 2.5 mg/mL A3G_fl_ in a 1 to 2 molar ratio with CCdZ-T_30_-oligo in A3G_fl_-SAXS buffer at 25 °C (50 mM phosphate, pH 6.0, 200 mM NaCl, 2 mM β-ME, 5% glycerol, 200 µM Na_2_-EDTA). In the text, the descriptor C for complex is added to distinguish these species from those identified in SAXS data of A3G_fl_ alone.

To study A3G_fl_ in complex with ssDNA, SEC-SAXS experiments were performed on A3G_fl_ in the presence of CCdZ-T_30_-oligo. Like the ligand-free A3G_fl_ sample, the sample was not homogeneous, and the eluents were not well resolved. Focusing on the elution profiles, the *R*g trace was observed to be variable across the protein elution peaks, indicating that multiple A3G_fl_ species were present in the sample (*R*g ~ 30–54 Å across 460–590 s, Figure 4A).

**Figure 5:**
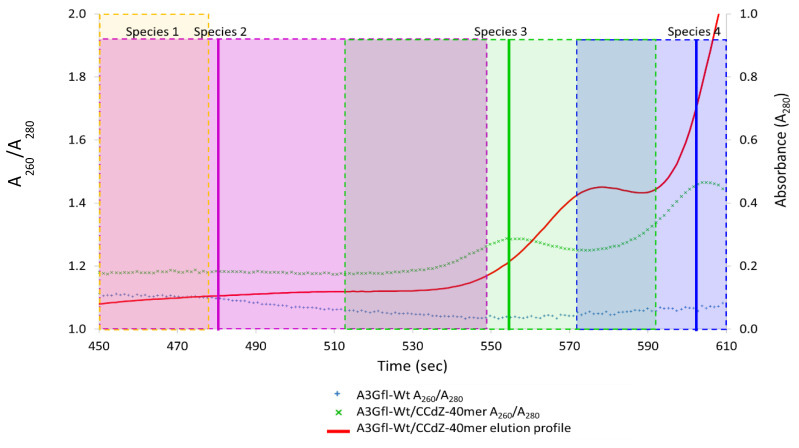
Ratio of A260/A280 to assess the presence of DNA in elution of the A3G_fl_/CCdZ-T30-oligo sample. The A260/A280 ratio of the A3G_fl_ (blue) and A3G_fl_/CCdZ-T30-oligo (green) samples (abbreviated as CCdZ-40mer in the figure), overlaid with the A280 elution profile of the A3G_fl_/CCdZ-T30-oligo sample (red) to display the boundaries of each species (Species-1, -2, -3 and -4). The peak maxima for the deconvoluted data are shown with a solid line; the peak width with a dotted rectangle and shaded box. Note: Peak widths for Species-1 and -4 extend to the left and to the right of the chromatograph, respectively. The color scheme is the same as in Figure 4. The overlap between the scattering of species results in the mixed colors.

In contrast, *R*g remained constant over the region where the oligo eluted (*R*g ~ 33 Å, from 597 to 645 s, Figure 4A), very similarly to the CCdZ-T_30_-oligo by itself. When we compared the absorbance maximum of the eluted ssDNA peak from this sample (A_280_ ~ 1.02, Figure S6C) to that of ssDNA-only control sample (A_280_ ~ 1.53, Figure S6B), we found that the absorbance decreased by ~30 %. This decrease was complemented by an increase in the overall absorbance of the eluted protein fractions (compare Figure S6A,C), establishing that a protein–ssDNA complex had formed. The superposition of the elution profiles (Figure 4B) clearly shows the shift of protein signal to the lower molecular size (shift to the right). As apo A3G_fl_ existed in multiple conformations, it was unclear from the elution profile which A3G_fl_ form was in complex with the oligonucleotide (further detailed in Figure 5). Therefore, deconvolution was performed (Figures 4C, S7 and S8 in Supplementary Materials) to extract the scattering curves for each A3G_fl_ component from the A3G_fl_/CCdZ-T_30_-oligo sample. Four species were identified (Figure 4C), and then, the four 1D scattering curves of each component (Figure S9A in Supplementary Materials) were extracted (termed Species 1–4).

Initially, we established using the double log plot [53] that all the derived scattering curves contained only one scattering species (see Figure S9B in Supplementary Materials). The Kratky plots of Species 1–3 presented characteristic bell-shaped peaks at low *q*, implying that the species were globular, though the structures also had flexible regions, as indicated by higher *q* data not completely returning to baseline (see Figure S9C in Supplementary Materials). These results were, again, consistent with the skewed shape of the *P*(*r*) curve from a standard bell-shaped curve, implying these components had elongated shapes (see Figure S9D in Supplementary Materials).

Interestingly, the Kratky plot of Species-4 had a broad peak that downturned at low *q*, as observed for DNA alone (see Figure S9C in Supplementary Materials). Due to the low abundance of Species-1, as indicated by its low signal intensity (Figure 4C), no accurate parameters could be derived for this species (see Figure S9B insert in Supplementary Materials). Therefore, we focused on deriving the structural information for Species-2, -3 and -4, as listed in Table S5 (Supplementary Materials).

### 3.6. Analysis of Species-2 from A3G_fl_/CCdZ-T_30_-Oligo SEC-SAXS

Based on the estimated MW, we deduced that Species 2 corresponded to an A3G_fl_ tetramer made up of four two-domain subunits (see Table S5, Supplementary Materials). The size (MW, *R*g, *D*max, see Table S5) of this species was slightly larger (estimate of 4.9 for free protein subunits) than that of the ligand-free tetrameric A3G_fl_ species (estimate of 4.4 free protein subunits, see Table S1 in Supplementary Materials), indicating that this component was potentially in complex with an oligonucleotide. To verify this notion, the A260/A280 ratio of the A3G_fl_ sample was compared to that of the A3G_fl_/CCdZ-T30-oligo sample, as described earlier [45]. Consistent with this notion, this A260/A280 ratio was larger than the ratio for the ligand-free protein across the entire elution profile, as displayed in Figure 5, illustrating that the A3G_fl_ Species-2 (A3G_fl_ tetramer) and other species (Species-1 and -3) elute along with the ssDNA. This signified that each of these species is a complex of A3G_fl_ with the CCdZ-T30-oligo. Species-4 is CCdZ-T30-oligo.

**Figure 6:**
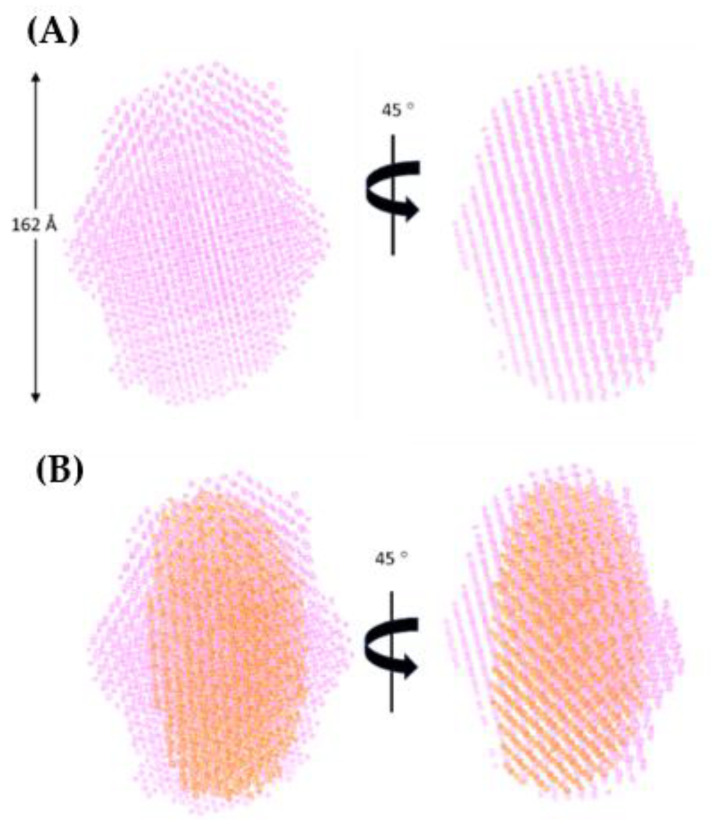
Envelope models of the A3G_fl_ tetramer with and without CCdZ-T30-oligo. (**A**) Envelope model of the A3G_fl_ tetramer/CCdZ-T30-oligo (purple) generated under P2 symmetry (DAMMIF, ATSAS 2.8.3 suite), with *D*max ~ 162 Å and *R*g ~ 47 Å. (**B**) A3G_fl_ tetramer/CCdZ-T30-oligo envelope model superimposed with ligand-free A3G_fl_ tetramer envelope model (orange).

Based on the differences between the MW of Species-2 (MW ~ 227 kDa, Table S5) and the ligand-free A3G_fl_ tetramer (MW ~ 203 kDa, Table S5), we can deduce, tentatively, that two oligonucleotides (24 kDa ~ two 12 kDa CCdZ-T30-oligos, Table S3) form a complex with the A3G_fl_ tetramer. To find the 3D shape of this species, ab initio shape reconstruction was performed using *P*2 symmetry to allow a tetrahedral or flattened tetrahedral arrangement. A mean NSD score of 0.557 for the averaged envelope model was derived, which indicated very good self-consistency for the ensemble (acceptable NSD ≤ 0.8) [63] (Table S6). The envelope model was somewhat ellipsoidal, as illustrated in Figure 6A. The envelope model of the previously identified ligand-free A3G_fl_ tetramer (Figure S4 in Supplementary Materials) fits well inside this envelope, establishing that the tetrameric arrangement observed for ligand-free A3G_fl_ is preserved on binding CCdZ-T30-oligo. However, for this A3G_fl_ tetramer with the CCdZ-T30-oligo, two noticeable regions of electron density appear on either side of the envelope of the ligand-free A3G_fl_ tetramer, as shown in Figure 6B. These regions indicate locations for the binding of two oligonucleotides, confirming earlier interpretations, whereby the A3G_fl_ tetramer (Species-2) complexes with two CCdZ-T30-oligos in solution, under our conditions.

Upon binding of the oligonucleotides to the A3G_fl_ tetramer, we also see that the protein envelope elongates slightly in comparison to the ligand-free form (Figure 6B), which is consistent with a 17 Å increase in *D*_max_, as well as a substantial increase in *R*g (see Table S5 Species-2 in comparison to Table S1). The action of binding the pair of CCdZ-T_30_-oligos to the tetrameric protein appears to cause modest structural rearrangements in the A3G_fl_ tetramer, leading to the observed elongation of the molecule. The two CCdZ-T_30_-oligos appear to be closely associated with and flattened onto the surface of the A3G_fl_ tetramer, but equally, the arms on either side of the dZ bound into the active site of two of the four CTDs of A3G_fl_ could be sampling many conformations and hence be undetectable by SAXS. However, the data do not permit distinguishing the two possibilities. Moreover, the tetrameric arrangement may be perturbed upon DNA binding.

### 3.7. Model-Free Analysis of SEC-SAXS Data for Species-3 and -4 from A3G_fl_/CCdZ-T_30_-Oligo

The parameters shown in Table S5 indicate that Species-3 is monomeric A3G_fl_ with one DNA bound (ratio of the MW from the Porod volume [53] to the mass of monomer with one DNA is 1.2, Table S5). As in the case of the free A3G_fl_ tetramer, the MW derived from the Porod volume is slightly higher than calculated and may be attributed, at least in part, to strongly associated water molecules. The retention time in SEC and the SAXS-derived parameters, along with the overall shapes of the plots derived for Species-4, are very similar to those of CCdZ-T30-oligo only, with the exception of MW from the Porod volume, indicating that Species-4 is a free CCdZ-T30-oligo. The Porod volumes are unreliable for non-globular flexible macromolecules, such as DNA.

### 3.8. Modeling Species-3 of A3G_fl_/CCdZ-T_30_-Oligo as a Monomer with DNA

**Figure 7:**
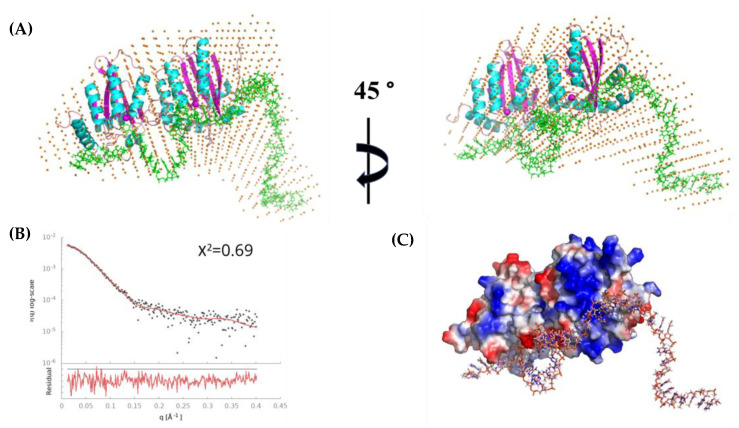
The model of CCdZ-T30-oligo in complex with monomer of A3G_fl_ (Species-3). (**A**) Fit of model with SAXS-derived envelope (orange dots)_in two orientations. The CTD of A3G_fl_ is on the left, the NTD is on the right; helices are shown in cyan, beta strands in magenta, loops in orange; the ssDNA is shown in green. (**B**) Fit (red solid line) of model with the SAXS profile (black dots), with residuals shown below. (**C**) The surface of the protein colored according to the charge distribution (red, negative; blue, positive) with CCdZ-T30-oligo superimposed (same orientation as right-hand frame of (**A**). The modeling was performed with PyMol (https://pymol.org/2/ (accessed on 18 August 2022)) using (1) the homology model (developed with YASARA (http://www.yasara.org/ (accessed on 18 August 2022)), using wild-type human sequence modeled onto full-length monomer from 6P40 [33], (2) the catalytically active C-terminal domain of A3G in complex with ssDNA 6BUX34 and (3) one DNA strand from B-DNA. The A3G-CTD/DNA complex (derived from 6P40) was used instead of free CTD in A3G_fl_ structure, and the single-stranded DNA was elongated from 9 nucleotides in the original structure to 40 nucleotides in our experiments. Zinc atoms in active and pseudo-active sites are shown as magenta spheres in (**A**).

The averaged envelope model for monomeric Species-3 (Figure 7A) resulted in a mean NSD score of 0.75 (acceptable NSD ≤ 0.8), indicating that the averaged model was acceptable (see Table S6 in Supplementary Materials). Therefore, we used this envelope for the modeling (Figure 7 and Figure S10 in the Supplementary Materials) of the A3G_fl_ monomer in complex with the DNA. The modeling was based on combining the homology model with the wild-type human A3G sequence based on the full-length monomer of A3G_fl_ from 6P40 [33] (Figure 7), the catalytically active C-terminal domain of A3G complexed with ssDNA (A3G-CTD/DNA, 6BUX [34]), and the remaining DNA was used to fit the envelope. This structural model for the full-length A3G–ssDNA complex gave an excellent fit with the SAXS data (χ^2^ = 0.69) with a random distribution of residuals, in sharp contrast to the poor fit of the DNA-free monomers (Figure S10A) showing more than 10 times worse χ^2^ value. Interestingly, this model for the A3G_fl_–ssDNA complex showed close proximity of the negatively charged phosphate backbone of the DNA with the positive patch on the N-terminal domain (NTD) (Figure 7C).

The modeling based on 6P3X [33] gave a very similar structure to that shown in Figure 7. Using the ssDNA monomers from 6WMA (Figure S10) resulted in a slightly worse fit with the SAXS profile (Figure S10C), but, interesting electrostatically, this model made more extensive contacts of ssDNA with the positive patches of the NTD. In this case, the different parts of ssDNA were interacting with the positive patches of NTD. From this modeling, we suggest that, on DNA binding, the protein can rearrange from one conformation to the other, and different positive patches encounter DNA. The confirmation of our models will require the higher-resolution methods, such as cryo-EM. Nevertheless, our low-resolution models give interesting insights. In the model shown in Figure 7, the pseudo-catalytic site of the N-terminal domain is interacting with the 3′ end of DNA, leading to the testable hypothesis that the NTD may recognize the CCC motif, allowing jumping and inter-segmental transfer.

Overall, the comparison between the deconvoluted SAXS profiles of Figure 1B and Figure 4B shows that in the presence of the DNA, the dimers disappear, and the monomeric state is much more prominent. The absence of a dimeric species (and the existence of tetrameric species) in the presence of a 40-mer ssDNA indicates the existence of (at least) two different interfaces, one of which competes with DNA and the other of which does not; namely, the dimeric interface that hides access to the active site should be outcompeted by DNA binding, but the tetrameric interface should be much less affected by it.

### 3.9. Model of Free A3G_fl_ Tetramer Based on Disappearance of Dimers and Preservation of Tetramers in the Presence of DNA

**Figure 8:**
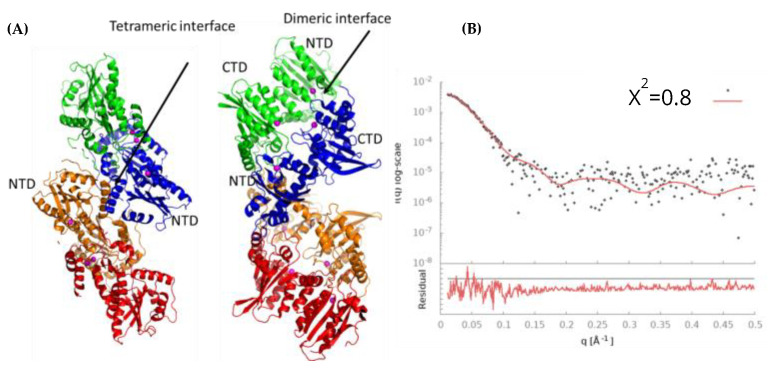
Tetramer model (**A**) featuring dimer and tetramer interfaces and fitting of this model (red solid line) with the SAXS scattering pattern (black dots) with residuals shown below (**B**). The right-hand image in (**A**) is rotated 90° clockwise about the line running from top to bottom to highlight the dimer interface. The dimer interface is that observed between pairs of A3G_fl_ (6P40), and the tetramer interface is that observed between the NTD of pairs of A3G_fl_ in 6P3X/Y/Z. Each monomer has its own color. The dimers of 6P40 were duplicated and overlaid with the 6p3x interface to produce the tertiary interface.

We now sought to understand the conundrum, whereby the tetramers of A3G_fl_ bind ssDNA, but the dimers of A3G_fl_ disappear in the presence of ssDNA. The inspection of the available pdb structures shows that full-length A3G from the Macaque monkey (6P40 [33]), which has a near-identical sequence to human A3G_fl_, dimerizes through the DNA-binding interface of the C-terminal domain and the putative DNA-binding interface of the N-terminal domain, suggesting that such a dimer would conflict with binding ssDNA. On the other hand, for a slightly different construct of the Macaque monkey A3G_fl_ (6P3X [33]; also 6P3Y and 6P3Z), the observed dimerization interface involves only the pairing of the NTD and, in contrast, does not occlude the active CTD sites of the dimer. We built the tetramer using two 6P40 dimers, with the tetramer interface corresponding to the 6P3X dimer interface. The fit of this tetramer model (Figure 8) with the SAXS scattering profile is remarkably good (χ^2^ = 0.69), especially as it was not based on the simple packing of molecules into the 3D reconstructed envelope (Figure S4 and Figure 6B). The radius of gyration calculated for this model is 41.7 Å, which is insignificantly different to those experimentally derived values of 41.5 ± 1.5 Å (from Guinier analysis) or 42.0 ± 1.0 Å (from *P*(*r*) analysis) (see Supplementary Materials, Table S1). The maximal length of this model is 148.5 Å compared to that derived from the *P*(*r*) plot of 145.9 Å (Table S1) and that derived for the envelope reconstruction of ~150 Å. Interestingly, fitting a tetramer incorporating two distinct crystallographically observed dimeric interfaces (Figure 8) gave a superior fit with the envelope and the SAXS pattern (χ^2^ = 0.80) than a simple packing of four monomeric A3G_fl_ molecules into the envelope (χ^2^ = 1.17).

Our tetramer model (Figure 8A) shows that the dimer interface of 6P40 is in complete overlap with the DNA-binding interface (Figure 7). Interestingly, in the dimer interface, both NTDs interact with the CTDs of another monomer (Figure 8A). The tetramer interface based on 6P3X, on the other hand, is built through NTD–NTD interactions. A human A3G_fl_ with mutations F126A/W127A to the NTD was shown to produce monomeric A3G_fl_ in solution [27]. Those residues form part of both the dimer and tetramer interfaces [33] (Figure 8) and are involved in the interaction with DNA according to our A3G_fl_–ssDNA model (Figure 7). Those mutations drastically reduce the jumping behavior of A3G_fl_ [27] and, according to our model, should affect the recognition of the CCC motif by the NTD, and therewith, affect the jumping.

## 4. Discussion

Here, using solution-state SAXS data, we report the structural models for the ligand-free tetrameric association of full-length APOBEC3G (A3G_fl_) and for A3G_fl_ in complex with single-stranded DNA (ssDNA). These are the first models for the binding of ssDNA to a two-domain APOBEC3 enzyme. Specifically, a structural model was derived for the interaction of monomeric A3G_fl_ with a 40-mer oligo, CCdZ-T30-oligo, which contains the inhibitor 2′-deoxyzebularine near the 5′-end. A prior SAXS study on A3G_fl_ was performed using the technology then available, in batch mode without size-exclusion chromatography, which obscured the presence of multiple oligomeric associations of A3G_fl_ species, both in the absence and presence of ssDNA [55]. We found the Guinier plots, eschewed in the previous study, essential for assessing the homogeneity and lack of aggregation of the species partially separated by size-exclusion chromatography (SEC) immediately prior to SAXS measurements from which the scattering of individual species was best extracted by Gaussian decomposition.

### 4.1. Multimeric Associations of A3G_fl_

Under our conditions, ligand-free two-domain A3G_fl_ eluted from the SEC column in multiple oligomeric forms, consistent with the published studies [41]. Scattering was dominated by higher-than-dimer oligomeric forms, scattering from monomeric and dimeric species was present, but in relatively low quantities, which precluded detailed analysis (Figure 1B). These various multimeric states have been proposed to regulate not only deamination-dependent but also deamination-independent functions of A3G proteins [18,41,64,65]. Using SAXS-derived parameters, we identified an A3G_fl_ tetramer as the most prominent scattering species. This species is formed from four two-domain A3G_fl_ molecules that SAXS data indicate are associated together in a compact formation (MW ~ 200 kDa). The structure of this tetramer was elucidated, based in part on the changes in distribution of oligomeric states upon adding the DNA, and in a larger part, on the recently published atomic-resolution A3G_fl_ dimer structures, which featured two distinctly different dimerization interfaces [32,33]. The generated A3G_fl_ tetramer model fits very well with our A3G_fl_ scattering data and, correspondingly, represents the A3G_fl_ tetrameric structure in solution. A key observation was that one dimerization interface hid the substrate-binding surfaces. The other dimerization interface, labeled the tetramer interface (Figure 8A), left the substrate-binding surfaces accessible. The substrate-binding surface giving access to the Zn^2+^ active site was observed in the case of the catalytically active C-terminal domain (CTD) but inferred for the catalytically inactive N-terminal domain (NTD).

In the presence of the CCdZ-T30-oligo, the monomeric A3G_fl_ species becomes much more prominent, while the dimers and putative 8-mers dissociate completely (see Figure 1 and Figure 4). Dimers are converted into monomers due to interactions with the ssDNA, and all our eluted A3G_fl_ species were identified to be in complex with ssDNA, as indicated upon examination of the A260/A280 ratio (Figure 5). Significantly, with respect to the interaction of A3G_fl_ with 40-mer ssDNA, the interface that occludes the ssDNA binding site (that of 6P40) is substantially more extensive and has a much more favorable free energy of association than the other dimerization interface (that of 6P3X). As calculated by PISA [66], respectively, the buried surface area is 9.5% compared to 3.8% of the total surface area, and the solvation free energy gain is −90 kJ mol^−1^ compared to −59 kJ mol^−1^. From this, we propose that the binding of negatively charged ssDNA, bearing dC or dZ in a CC(C/dZ) motif, displaces the interface interactions between the A3G_fl_ molecules of the dimeric and 8-mer oligomerizations of A3G_fl_. Interestingly, an increase in the relative amount of monomeric species was also seen in A3B-CTD upon the addition of ssDNA [45]. With the dimer interface of A3G_fl_ separated, the less extensive tetramer interface becomes less stable. The apo-A3G_fl_ tetramer, however, does form a complex, but with just two CCdZ-T30-oligos. A model structure was difficult to elucidate, as the tetrameric structure may rearrange upon the DNA binding. Moreover, this DNA binding should be non-specific (in case the tetramer does not completely rearrange), as the substrate-binding sites, especially the catalytically active site in the C-terminal domain, are buried in the dimerization interfaces. The bulge in electron density observed in the envelope reconstruction, relative to the ssDNA-free tetramer (Figure 6B), coincides with the region of interaction of a pair of NTDs, suggesting that the negatively charged ligand, CCdZ-T30-oligo, is interacting with the NTDs, each of which is much more positively charged than the CTDs. This interpretation contradicts a previously proposed model, whereby tetrameric species are more active than monomeric and dimeric ones [42], but our interpretation is in line with the similar catalytic activity observed for a monomeric mutant and wild-type A3G [27] and with the oligomer disruption shown by optical tweezers [19].

### 4.2. Modeling the Interaction of CCdZ-T_30_-Oligo with Monomeric A3G_fl_

An A3G_fl_ monomer complexed with CCdZ-T30-oligo was observed and characterized, revealing the first full-length double-domain catalytically active A3 model structure in complex with ssDNA. Our A3G_fl_-40mer ssDNA model, based on the reported A3G_fl_ crystal structures [33] and on the extended DNA from an ssDNA complex with the CTD of A3G_fl_ [34], gave a remarkably good fit with the observed (deconvoluted) scattering for this species. Our analysis provides a structural explanation for the observation of A3G_fl_ cycling between high molecular weight assemblies and monomeric-ssDNA species, as A3G_fl_ searches and deaminates cognate ssDNA [19], as dimerization, via paired NTD and CTD interactions, and DNA binding are mutually exclusive, according to our models. In addition, higher activity has been reported [18] for of monomeric A3G_fl_ as compared to high-order species, where active sites are buried.

The model shows the interaction of ssDNA with both domains (NTD and CTD) (Figure 7A and Figure S10B). The models show a DNA looping roughly consistent with the 1 nm minimal loop size suggested by the optical tweezers experiments [19]. Interestingly, the DNA in the model shown in Figure 7A is close to the pseudo-catalytic zinc-containing site of the NTD. As our 40-mer has only one recognition motif (CCdZ), the NTD is likely binding somewhat non-specifically to the ssDNA. We can speculate that a second CCC motif could be bound to the NTD pseudo-active site, which should result in a much larger looping of the DNA Such looping was inferred from the experiments performed with optical tweezers [19] and consistent with DNA bending seen in single-molecule FRET [28]. We propose that the conformation seen in our model in Figure 7 is well suited to explain the jumping [24] and inter-segmental transfer [26] on ssDNA with multiple CCC motifs. This model suggests that the NTD can recognize the CCC motif and thereby contribute to the quick search on the longer DNA through jumping, sliding [22] and inter-segmental transfer [26]. The NTD residues shown to affect jumping [24] are interacting with or are close to the DNA in our model. The conformation relevant to sliding may be different and could be similar to the model in Figure S10B based on the 6WMA structure [32], as a mutation in helix 6 of the NTD [24] affects sliding, and helix 6 will be close to the DNA if 6WMA is used instead of 6P40 in the modeling (Figure S10B,D). Our modeling is also consistent with the interpretation that the observed 30-nucleotide “dead zone” [27] is the minimal number of nucleotides required to allow all the necessary structural rearrangements between the sliding and jumping modes on ssDNA required for efficient searching for CCC deamination motifs. This interpretation is supported by the fact that the processivity factor increases until 30-nucleotide separation between two CCC motifs is reached and is constant afterward up to 100 nucleotides [67]. Significantly, our models for the interaction of ssDNA with A3G_fl_ show that ~30 nucleotides lie close to the protein, with the remaining nucleotides of the 40-mer ssDNA projected away from the protein. Moreover, consistent with the direction of processivity, the residues 3′ of the CCC deamination motif (here, CCdZ) pass over the non-catalytic NTD. Put another way, the nucleotides in the 3′ end of ssDNA are necessary to allow a full interaction of A3G_fl_ with ssDNA for effective search for CCC deamination motifs on longer DNA. Our work shows that, at least in our conditions, ssDNA is interacting with both domains of A3G, as this interaction is necessary to reasonably fit the SAXS data (see Figure S11 and discussion in the Supplementary Materials). Based on chemical crosslinking [68], only the C-terminal domain of A3G was suggested for the DNA interaction in the model for A3G functional regulation by the RNA [69]. This model can be easily adapted to include additional DNA interaction with the N-terminal domain of A3G.

A further consideration to emerge from our structural models is the potential role of allostery in the binding of substrate to A3G_fl_. Noting the very different positioning of the NTD with respect to the CTD for human (PDB: 6WMA) [32] compared to rhesus monkey A3G_fl_ (PDB 6P40, 6P3X) [33], DNA binding to the NTD may cause a conformational change to the relative arrangement of NTD and CTD domains, which may help to better position dZ (or substrate cytosine) close to the CTD active site. Upon specific binding of the dZ into the CTD active site, the dZ is hydrated across the N3-C4 double bond and converted into an intermediate state of deamination, in which C4 is tetrahedral, and dZ remains bound to the protein [59,60]. The nucleotides flanking the target dZ stack on top of one another to stabilize the overall conformation, as observed in the single-domain A3–ssDNA complex structures [34].

## 5. Conclusions

Although intrinsically a low-resolution technique, our SAXS results provide cogent and coherent insight into the oligomerization of ligand-free full-length APOBEC3G and into the de-oligomerization occurring upon binding of single-stranded DNA bearing an inhibitor CCdZ motif that targets the catalytically active site of the C-terminal domain of A3G_fl_. A key result is that ssDNA binding to and dimerization of A3G_fl_ are mutually exclusive. Our modeling strongly suggests that the intermolecular interactions observed in the crystal structures of A3G_fl_ are maintained in solution. Moreover, the structural models developed provide an insight into a range of earlier biochemical studies. A somewhat unexpected mode of binding of a 40-mer inhibitor species, CCdZ-T_30_-oligo, to the catalytic and pseudo-catalytic sites gives a structural insight into the A3G_fl_ interaction with ssDNA and provides a structural basis for the hitherto unexplained jumping mode of action of A3G_fl_ on ssDNA substrates, as well as for the observed 30-nucleotide “dead zone” where at least 30 nucleotides located 3′ to the CCC motif are required for full, processive activity. An important role for the pseudo-catalytic N-terminal domain of A3G_fl_ and further confirmation of directionality in processivity is revealed by the 40-mer inhibitor CCdZ-T_30_-oligo being more potent than the 5′-tailed inhibitor T_30_-CCdZ-oligo or a short 9-mer oligomer in which the CCdZ motif is embedded. These results will help guide further structural studies and highlight the use of modified oligonucleotides for studies of active A3 in complexes with DNA.

## Data Availability

SAXS data are deposited in SASBDB under the codes: SASDMX6, SASDMY6, SASDMZ6, SASDM27.

## Supplementary Materials

The following are available online at https://www.mdpi.com/article/10.3390/v14091974/s1. Figure S1: Deconvolution of the SAXS data of the A3G_fl_; Figure S2: SAXS analysis of A3G_fl_ deconvoluted species; Figure S3: Correlation between log of the oligomeric state and size exclusion profile; Figure S4: Ab initio shape restoration of A3G_fl_ tetramer; Figure S5: SEC-SAXS analysis of the CCdZ-T_30_-oligo; Figure S6: SEC-FLPC profile comparison of apo A3G_fl_, CCdZ-T_30_-oligo (denoted in figure as CCdZ-40mer) and A3G_fl_/CCdZ-T_30_-oligo sample shows complex formation; Figure S7: SEC-SAXS analysis of sample of A3G_fl_/ CCdZ-T_30_-oligo (denoted in figure as CCdZ-40mer); Figure S8: Singular value decomposition (SVD) of sample of A3G_fl_ with CCdZ-T_30_-oligo; Figure S9: SEC-SAXS analysis of A3G_fl_/ CCdZ-T_30_-oligo deconvoluted species; Figure S10: Modeling of A3G_fl_/CCdZ-T_30_-oligo complex; Figure S11: Fit of 40-mer oligonucleotide to data, where only ten residues are in contact in the vicinity of the catalytically active C-terminal domain; Figure S12: Tetramer model shown in Figure 8 (main text) is superimposed with the A3G_fl_ tetramer envelope model (orange) shown in Figure S4; Table S1: SAXS structural parameters of the A3G_fl_; Table S2: SAXS fitting and modeling parameters of the A3G_fl_ sample; Table S3: SAXS structural parameters of CCdZ-T_30_-oligo; Table S4: SAXS fitting and modeling parameters of CCdZ-T_30_-oligo; Table S5: SAXS structural parameters of A3G_fl_ in presence of CCdZ-T_30_-oligo; Table S6: SAXS ab initio modeling parameters of A3G_fl_ in presence of CCdZ-T_30_-oligo [70].
