# Supplementary material for: Small-Angle X-ray Scattering (SAXS) Measurements of APOBEC3G Provide Structural Basis for Binding of Single-Stranded DNA and Processivity
Source: Viruses. 2022 Sep 6;14(9):1974. doi: 10.3390/v14091974 (PMC9505750; doi:10.3390/v14091974)
Supplement: Supplementary file 1 [file viruses-14-01974-s001.zip › viruses-1815887-supplementary.pdf]

Supplementary Material

Small-angle X-ray scattering (SAXS) measurements of APOBEC3G provide structural basis for binding of single-stranded DNA and processivity

Fareeda M. Barzak [†], Timothy M. Ryan[*], Nazanin Mohammadzadeh [‖,2], Stefan Harjes [†], Maksim V. Kvach [†], Harikrishnan M. Kurup [†], Kurt L. Krause [§, ‡], Linda Chelico [‖], Vyacheslav V. Filichev* [†, ‡], Elena Harjes* [†, ‡], and Geoffrey B. Jameson* [†, ‡]

[†] School of Natural Sciences, Massey University, Private Bag 11 222, Palmerston North 4442, New Zealand, [‖] Department of Biochemistry, Microbiology, and Immunology, University of Saskatchewan, Saskatoon, Saskatchewan S7N 5E5, Canada, [*] SAXS/WAXS, Australian Synchrotron/ANSTO, 800 Blackburn Road, Clayton, VIC3168, Australia, [§] Department of Biochemistry, University of Otago, PO Box 56, Dunedin 9054, New Zealand, [‡] Maurice Wilkins Centre, University of Auckland, Auckland 1142, New Zealand.

[2] Current address: Medical Microbiology and Immunology Department, University of Alberta, Edmonton, Alberta, Canada, T6G 2R3

*Correspondence: G.B.Jameson@massey.ac.nz, e.harjes@massey.ac.nz, v.filichev@massey.ac.nz

SEC-SAXS analysis of apo A3G$_{fl}$: deconvolution of scattering data

The singular value decomposition (SVD) function was used to define the number of components in the sample (referred to as eigenvalues). Then the evolving factor analysis (EFA) method was utilized to define the boundaries and extract the scattering curves of each component. This analysis showed three potential components being present in the sample (frames 300-529, 352-546, and 398-612) (Supplementary Figure S1C). However, as the boundaries were significantly overlapped it was difficult to isolate the data of each scattering species. Moreover, the decomposition produced physically implausible negative values for scattering of components, and one component (EFA_3) clearly seems to comprise two components. Therefore, Gaussian decomposition analysis (US-SOMO HPLC-SAXS module) was performed by fitting Gaussian functions to produce five 1D-scattering curves (Figure S1D) [1-3].

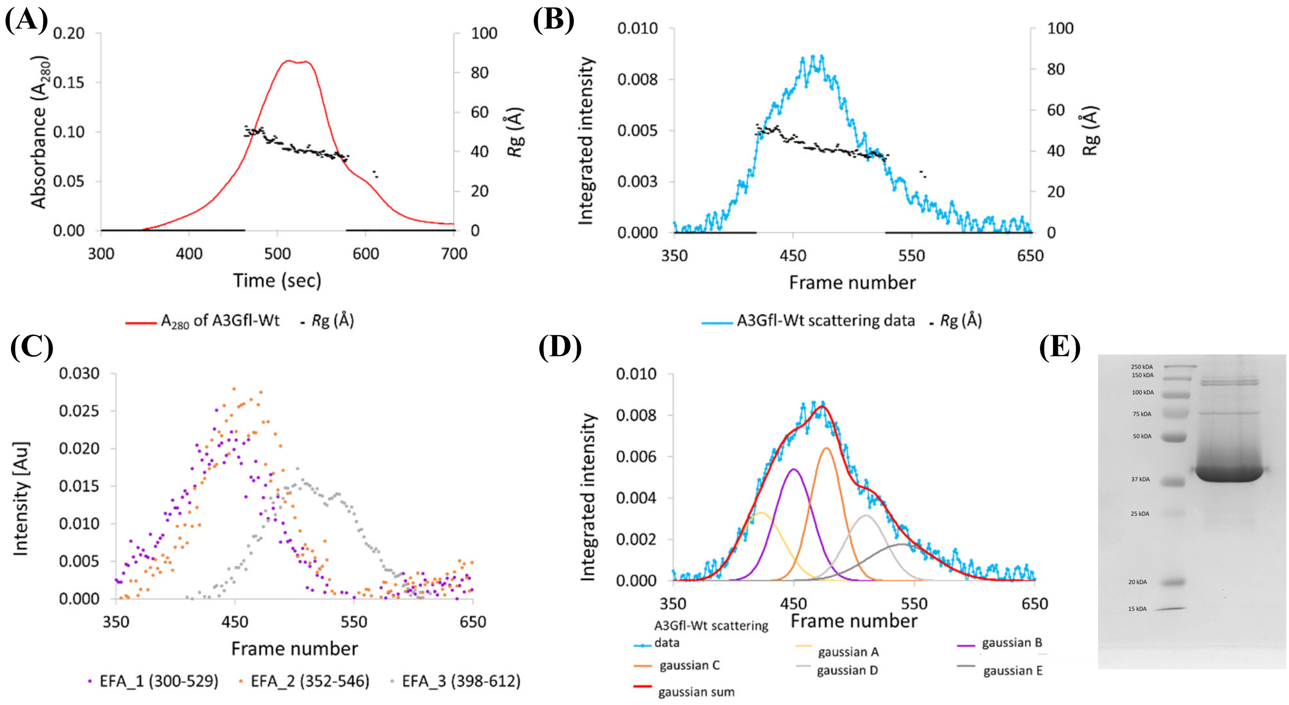

Figure S1. Deconvolution of the SAXS data of the A3G$_{fl}$.

SEC-SAXS analysis of A3G$_{fl}$. (**A**) SEC elution profile collected as A_{280} and (**B**) SAXS profile superimposed with *Rg* trace values. (**C**) EFA analysis using BioXTAS RAW. (**D**) Gaussian decomposition analysis using US-SOMO. Experiments were conducted at 25 °C using 2.5 mg/mL A3G$_{fl}$ in A3G$_{fl}$ SAXS buffer (50 mM phosphate pH 6.0, 200 mM NaCl, 2 mM β-ME, 5% glycerol, 200 μM Na_2-EDTA). (**E**) A Coomassie stained SDS-PAGE gel of the material that was applied to the SEC-co-flow column. 81 μg of purified protein is loaded on the gel.

For full-length A3G alone, as is clear from Figure S1C, the EFA analysis failed to reproduce the observed SAXS profile. Both the A_{280} elution profile and the SAXS profile clearly show a minimum of four components – noting also that the A_{280} profile scales linearly with particle volume, whereas the SAXS profile scales with particle volume-squared. It was only after deconvolution into five components that we noticed the remarkable linearity in a plot of log(oligomerisation) vs frame number/elution time (Figure S3), where from prior knowledge of tetrameric species we assumed, as the simplest possible explanation, that oligomerisation was multiples of two (ie, 1,2,4,8,16).

Gaussian deconvolution is appropriate as the dimer interfaces of 6P40 and 6P3X calculated by PISA (https://www.ebi.ac.uk/msd-srv/prot_int/cgi-bin/piserver) are both extensive and robust and thus reproportionation among the species of greatest interest, tetramer, dimer and monomer, during SEC is expected to be negligible. Significantly the dimer interface that occludes the ssDNA binding site (6P40) has the more extensive buried surface (9.5%) and the more favourable free-energy of association (−90 kJ mol^{-1}) than the other dimer interface (6P3X/Y/Z: respectively, 3.8% and −59 kJ mol^{-1}). Thus, for monomer-dimer-tetramer species, rapid exchange with consequent non-Gaussian peak shapes under SEC is highly unlikely.

.

SAXS analysis of apo $A3G_{fl}$: analysis of the scattering curves

The Guinier distribution analysis of $A3G_{fl}$-species B using data in the low q range resulted in an unsatisfactory fit to the linear regression, which compromised estimation of the Rg and $I(0)$ (see Figure S1B insert).

A*b initio* shape restoration (DAMMIF*,* ATSAS 2.8.3 suite [4]) was performed with a $P2$ symmetry constraint to allow a tetrahedral or flattened tetrahedral arrangement around a two-fold rotational symmetry axis. Modelling the molecular envelope without symmetry, or assuming four-fold symmetry, also gave a very similar shape; in the case of $P4$ symmetry, a slightly better NSD of 0.695 was obtained.

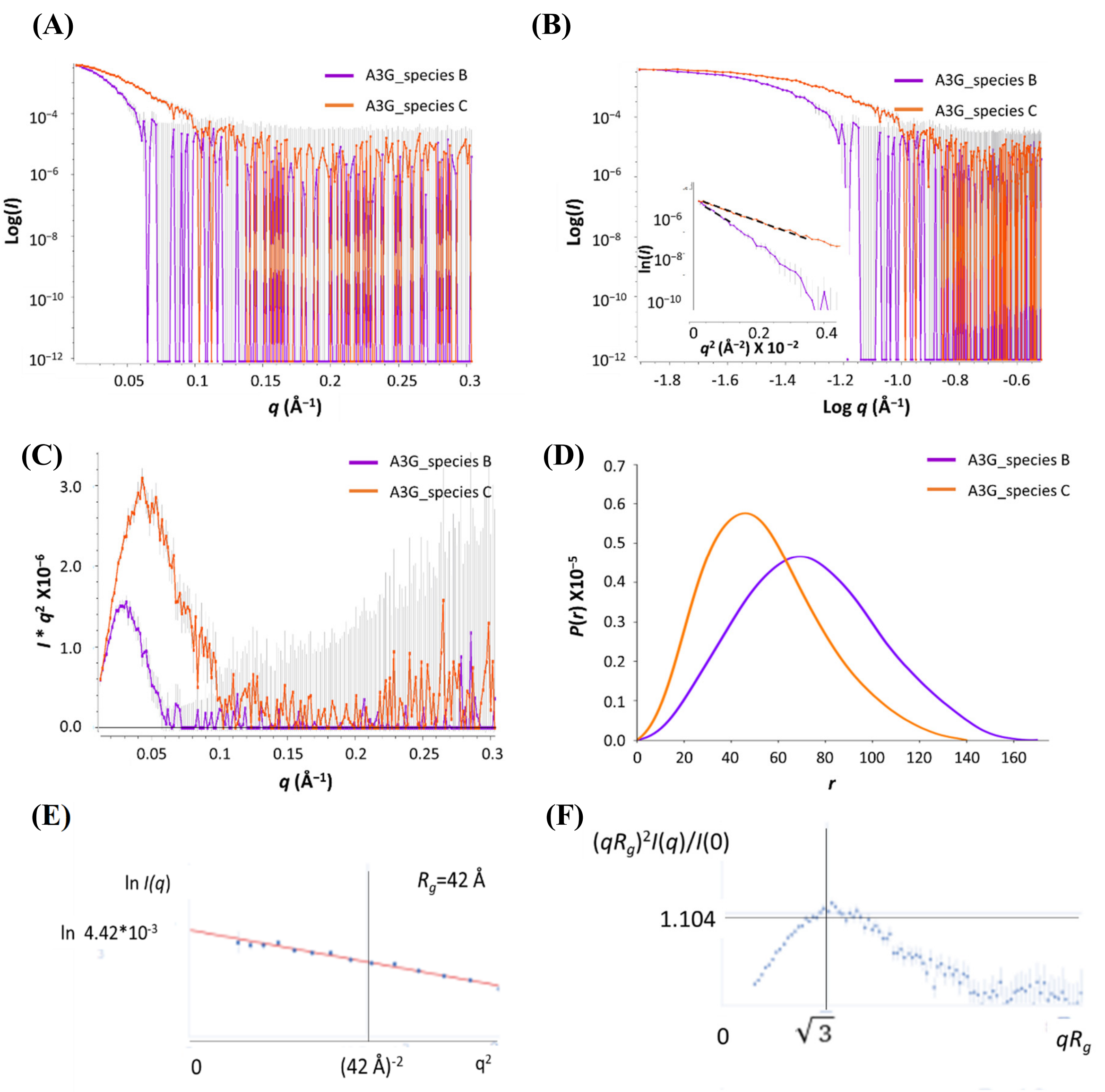

Figure S2. SAXS analysis of A3G$_{fl}$ deconvoluted species.

Analysis of the scattering data of the deconvoluted A3G$_{fl}$ species was conducted using several programs from the PRIMUSQT ATSAS 2.8.3 suite. (**A**) 1D-scattering curves of the species. (**B**) Double log plot with insert Guinier plot (full range going beyond $q{\cdot}R_g < 1.30$, compare to (E)). (**C**) Kratky plot. (**D**) $P(r)$ distribution plot. Experiments were conducted using 2.5 mg/mL of A3G$_{fl}$ in pH 6.0 SAXS buffer (see Figure S1). Note: precision in determining R_g for A3G$_{fl}$_species B is low because of limited number of points between camera-limited lower limit of q and the limit of the Guinier analysis $q{\cdot}R_g < 1.30$ as $R_g \sim 60$ Å. (**E**) Guinier plot for species C in the range to $q{\cdot}R_g < 1.30$ as $R_g \sim 42$ Å, q^2 is < 0.001 Å^{-2}. (**F**) R_g-normalized dimensionless Kratky plot for species C[5].

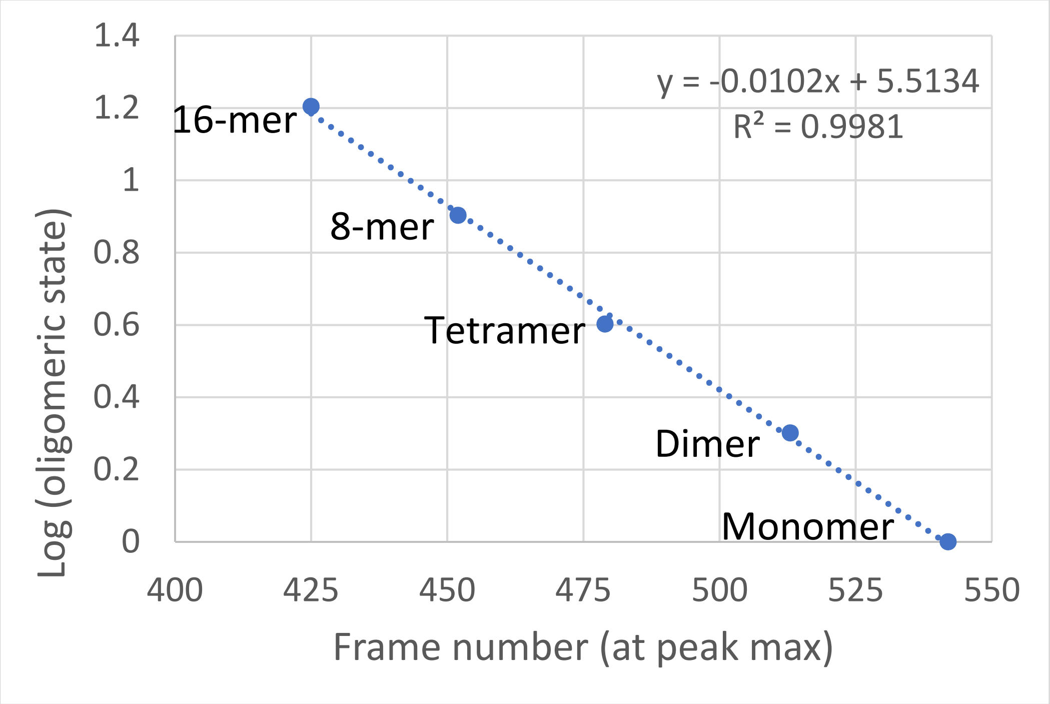

Figure S3: Correlation between log of the oligomeric state and size exclusion profile. The scattering frame number is equivalent to retention time at the maximum scattering intensity deconvoluted for a given species).[5]

Table S1. SAXS structural parameters of the A3G$_{fl}$.

Structural parameters	A3G_species C (A3G$_{fl}$ tetramer)
Guinier analysis	
$I(0)$ (cm^{-1})	0.0044 ± 0.0001
R_g (Å)	41.5 ± 1.5
q_{min} (Å^{-1})	0.01245
$q \cdot R_g$ max	1.3
Coefficient of correlation (R^2)	0.95
P(r) analysis	
$I(0)$ (cm^{-1})	0.0044 ± 0.0001
R_g (Å)	42.0 ± 1.0
D_{max} (Å)	145.9
q range (Å^{-1})	0.01245 - 0.34030
Quality estimate	0.85
Porod volume (Å^3)	346,000
MW (Porod Volume*0.6) (Da) (ratio to expected subunit 46,408 Da)	202,600 (4.4)

Table S2. SAXS fitting and modelling parameters of the A3G$_{fl}$ sample

Modelling parameters	A3G_species C
Ab initio restoration	
DAMMIF [a]	
q range ($Å^{-1}$) for fitting	0.01245 - 0.34030
Symmetry	*P*2
NSD (standard deviation)	0.789 (0.254)
Resolution (from SASRES) (Å)	62 ± 5
**Structure modelling**	
q range for modelling ($Å^{-1}$)	0.01245 - 0.34030

[a] Default parameters, 10 calculations; NSD describes the normalized spatial discrepancy (NSD) score. [b]

A*b initio* shape restoration [4] was performed (Figure S4). The averaged low-resolution dummy model had an acceptable NSD value (quality factor) of 0.789 indicating that the models were in good agreement with experimental data (see Table S2). The envelope model exhibited an elongated rugby-ball shape, consistent with observations from the Kratky plot[6] and $P(r)$ plot[6] (see Figure S2C and S2D).

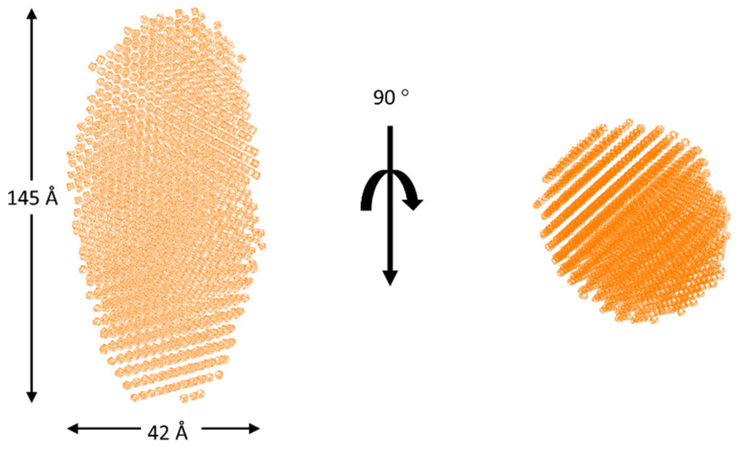

Figure S4. *Ab initio* shape restoration of A3G$_{fl}$ tetramer.
Envelope models generated using DAMMIF *(ATSAS 2.8.3 suite)* under *P*2 symmetry of A3G_Species C (tetramer) and further refined using DAMAVER and DAMFILT (*ATSAS 2.8.3 suite)*.

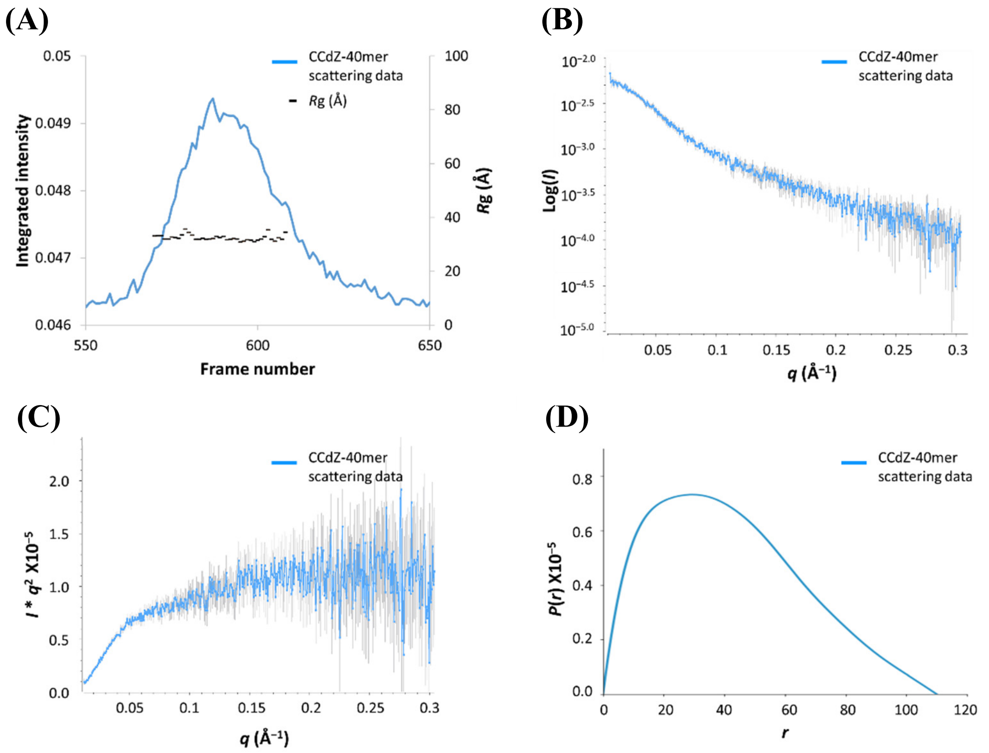

Figure S5. SEC-SAXS analysis of the CCdZ-T_{30}-oligo (denoted in figure as CCdZ-40mer oligo). SAXS analysis of the scattering data of CCdZ-T_{30}-oligo conducted using several programs from the PRIMUSQT *ATSAS 2.8.3 suite.* (**A**) SAXS profile with R_g trace values superimposed over profile. (**B**) Scattering curve. (**C**) Kratky plot. (**D**) *P*(*r*) distribution plot. Experiments performed using 5 mg/mL of CCdZ-T_{30}-oligo ssDNA in A3G_{fl} pH 6 SAXS buffer. Note: in main text CCdZ-40mer oligo is referred to more explicitly as CCdZ-T_{30}-oligo with full sequence ATTCCdZAATTT_{30}.

Table S3. SAXS structural parameters of CCdZ-T_{30}-oligo.

Structural parameters	CCdZ-T_{30}-oligo
Guinier analysis	
$I(0)$ (cm^{-1})	0.00530 ± 0.00012
R_g (Å)	32.0 ± 1.0
q_{min} ($Å^{-1}$)	0.0165
$q \cdot R_g$ max	1.3
Coefficient of correlation (R^2)	0.97
P(r) analysis	
$I(0)$ (cm^{-1})	0.005260 ± 0.000008
R_g (Å)	32.2 ± 0.3
D_{max} (Å)	118.0

q range (Å^{-1})	0.0165 - 0.2975
Quality estimate	0.78
Porod volume (Å^3)	20,700
MW (Porod Volume*0.6) (Da) (ratio to expected subunit 12,084 Da)	12,420 (1.03)
MW (from *I*(0) [14]) (Da) (ratio to expected subunit 12,084 Da)	13,150 (1.09)

Table S4. SAXS fitting and modelling parameters of CCdZ-T_{30}-oligo.

**Modelling parameters**	CCdZ-T_{30}-oligo
Ab initio restoration	
DAMMIF (default parameters, 10 calculations)	
q range (Å^{-1}) for fitting	0.0165 - 0.2975
Symmetry	*P*1
NSD (standard deviation)	0.875 (0.052)
Resolution (from SASRES) (Å)	36 ± 3
Structure modelling	
40mer ssDNA B-form model	
FoXS, Chi^2	2.96
CRYSOL, Chi^2	1.84

SAXS model of A3G$_{fl}$ in the presence of dZ-containing ssDNA

Initial EFA deconvolution estimated four components in the sample (see Figure S7B) with the following boundaries for each species (frames 385-526, 446-559, 485-583 and 477-590, Figure S7B). The components 2, 3, and 4 were major contributors to the scattering pattern, while component 1 was barely present (see Figure S7B). All-in-all the EFA decomposition was unconvincing. To further isolate the 1D-scattering curves of each component, Gaussian decomposition analysis (US-SOMO) was performed as described for apo A3G$_{fl}$. Four Gaussian functions were fitted to the data (Figure 4C), and then converted into four 1D-scattering curves of each component (termed Species 1-4) (Figure S9).

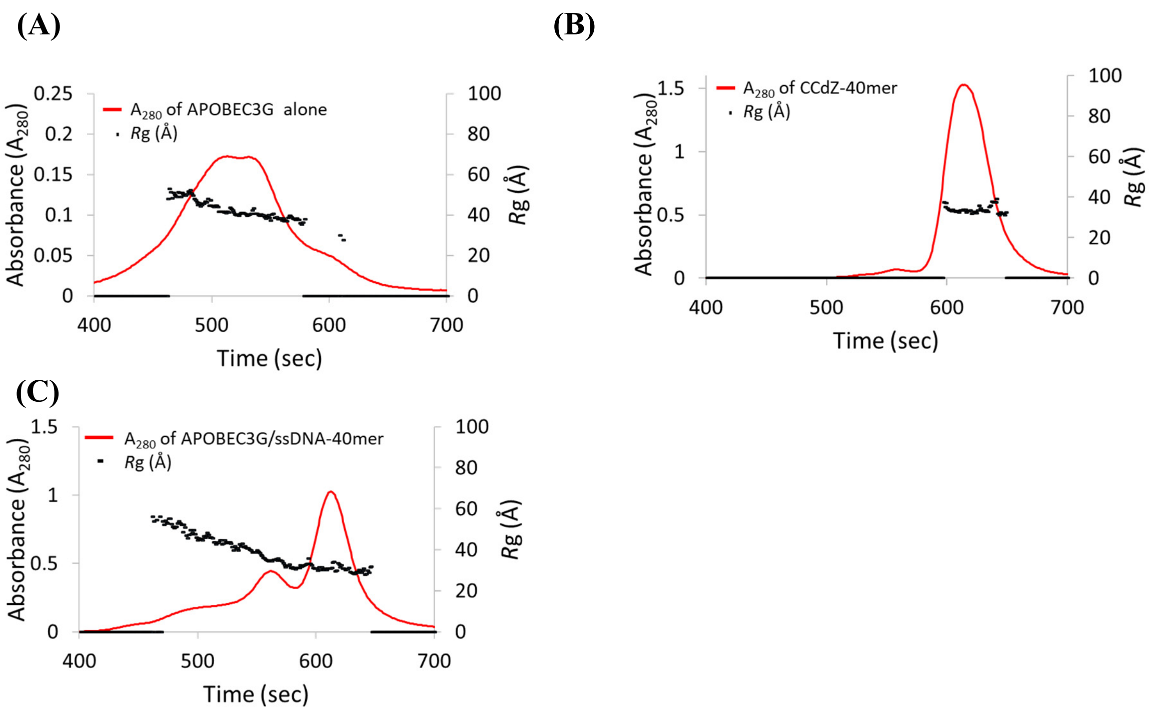

Figure S6. SEC-FLPC profile comparison of apo A3G$_{fl}$, CCdZ-T$_{30}$-oligo (denoted in figure as CCdZ-40mer), and A3G$_{fl}$/CCdZ-T$_{30}$-oligo sample shows complex formation.

SEC-FPLC elution profiles of A3G$_{fl}$ alone (**A**), of CCdZ-T$_{30}$-oligo (**B**) and of A3G$_{fl}$ with CCdZ-T$_{30}$-oligo. (**C**) at a 1 to 2 ratio, with R_g trace values superimposed. Experiment conducted using 2.5 mg/mL A3G$_{fl}$ in a 1 to 2 molar ratio with CCdZ-T$_{30}$-oligo in A3G$_{fl}$-SAXS buffer at 25 °C (50 mM phosphate pH 6.0, 200 mM NaCl, 2 mM β-ME, 5% glycerol, 200 µM Na$_2$-EDTA).

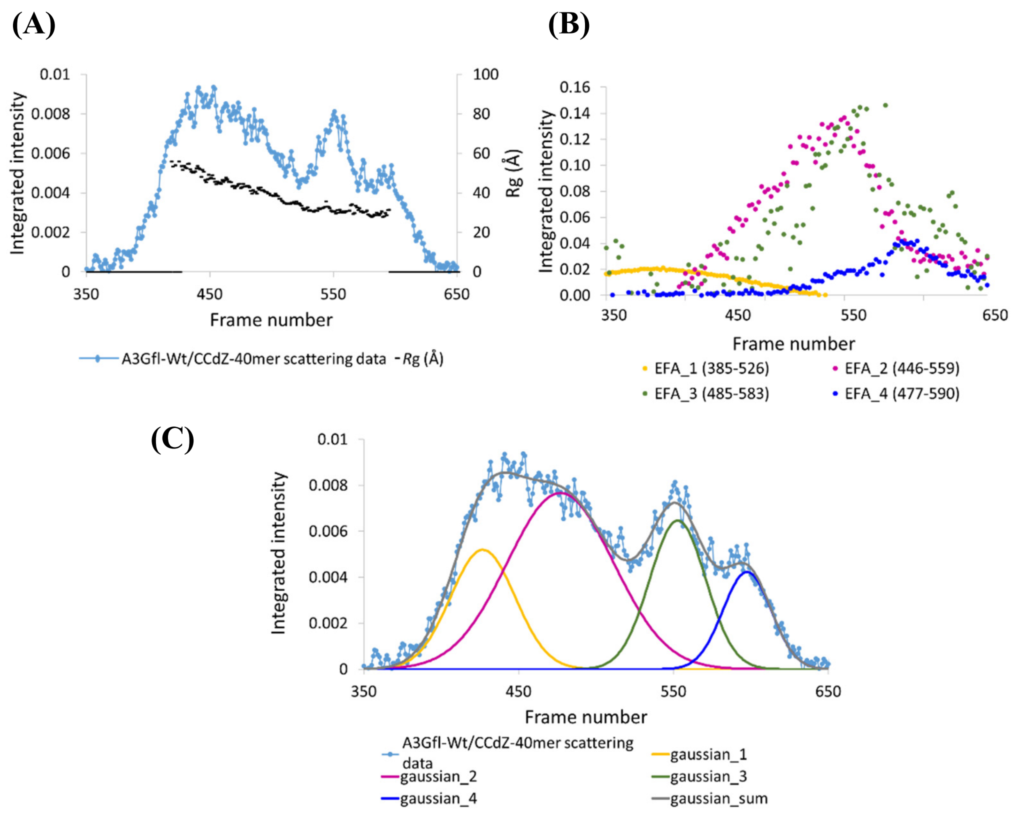

Figure S7. SEC-SAXS analysis of sample of $A3G_{fl}$/ CCdZ-T_{30}-oligo (denoted in figure as CCdZ-40mer).

(**A**) SAXS profile with *R*g trace values superimposed over profile. (**B**) EFA analysis using BioXTAS RAW. (**C**) Gaussian decomposition analysis using US-SOMO. Experiment conducted using 2.5 mg/mL $A3G_{fl}$ in a 1 to 2 molar ratio with CCdZ-T_{30}-oligo in $A3G_{fl}$-SAXS buffer at 25 °C (50 mM phosphate pH 6.0, 200 mM NaCl, 2 mM β-ME, 5% glycerol, 200 µM Na_2-EDTA).

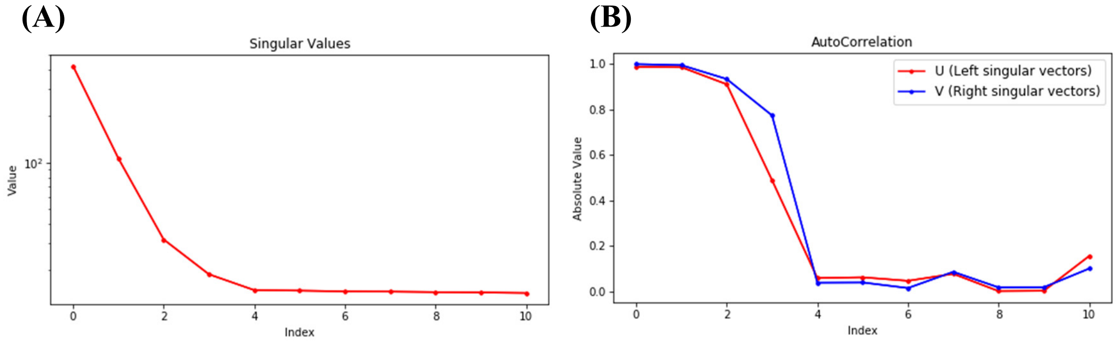

Figure S8. Singular value decomposition (SVD) of sample of $A3G_{fl}$ with CCdZ-T_{30}-oligo.

SAXS profile assessed using SVD/EFA BioXTAS RAW [6]. (**A**) SVD showing the eigenvalues where the index represents number of eigenvalues equivalent to number of components in scattering sample. Top panel represents SVD showing an eigenvalue of four. (**B**) Autocorrelation between the singular vectors (blue and red lines), indicating that the singular values are not too variable.

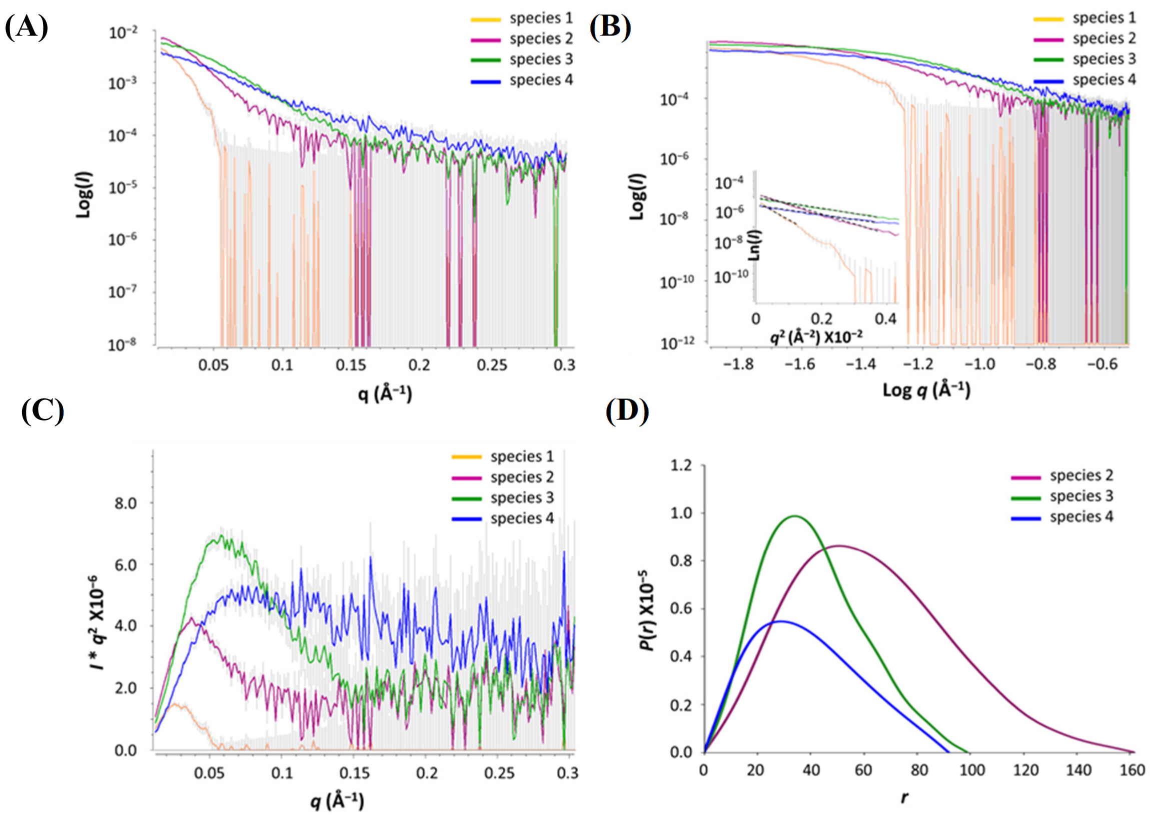

Figure S9. SEC-SAXS analysis of A3G$_{fl}$/ CCdZ-T$_{30}$-oligo deconvoluted species.

Analysis of the scattering data of the A3Gfl/ CCdZ-T$_{30}$-oligo deconvoluted species was conducted using several programs from the PRIMUSQT *ATSAS 2.8.3 suite* [8]. (**A**) 1D-scattering curves of each species. (**B**) Double log plots with insert of a Guinier plots. (**C**) Kratky plots. (**D**) *P*(*r*) distribution plots. Experiments were conducted using 2.5 mg/mL of A3G$_{fl}$ in SAXS buffer (see caption to Figure S7).

Table S5. SAXS structural parameters of $A3G_{fl}$ in presence of CCdZ-T_{30}-oligo.

Structural parameters	Species-2	Species-3	Species-4
Guinier analysis			
$I(0)$ (cm^{-1})	0.00812 ± 0.00018	0.00492 ± 0.00093	0.00261 ± 0.00092
R_g (Å)	47.0 ± 1.4	33.6 ± 1.2	29.8 ± 1.7
q_{min} (Å^{-1})	0.0139	0.0183	0.0183
$q{\cdot}R$g max	1.3	1.3	1.3
Coefficient of correlation (R^2)	0.98	0.45	0.57
P(r) analysis			
$I(0)$ (cm^{-1})	0.00834 ± 0.00012	0.00496 ± 0.00069	0.00244 ± 0.00044
R_g (Å)	48.4 ± 0.6	32.7 ± 0.5	30.4 ± 0.4
D_{max} (Å)	162.1	100.1	92.9
q range (Å^{-1})	0.0139 - 0.3052	0.0183 - 0.3052	0.0183 - 0.3052
Quality estimate	0.89	0.80	0.79
Porod volume (Å^3)	379,000	117,000	62,700
MW (Porod Volume*0.6) (Da) (ratio to expected subunit size 46,408 Da (protein) +12.1 Da (DNA))	227,400 (tetramer + 2 DNA molecules)	70,200 (1.2)	37,620

Table S6. SAXS *ab initio* modelling parameters of $A3G_{fl}$ in presence of CCdZ-T_{30}-oligo.

Modelling parameters	A3G_species-2	A3G_species-3	A3G_species-4
Ab initio restoration			
DAMMIF (default parameters, 10 calculations)			
q range (Å^{-1}) for fitting	0.0139 - 0.3052	0.0183 - 0.3052	0.0183 - 0.3052
Symmetry	P2	P1	P1
NSD (standard deviation)	0.557 (0.036)	0.750 (0.126)	0.559 (0.049)
Resolution (from SASRES) (Å)	39 ± 3	40 ± 3	35 ± 3

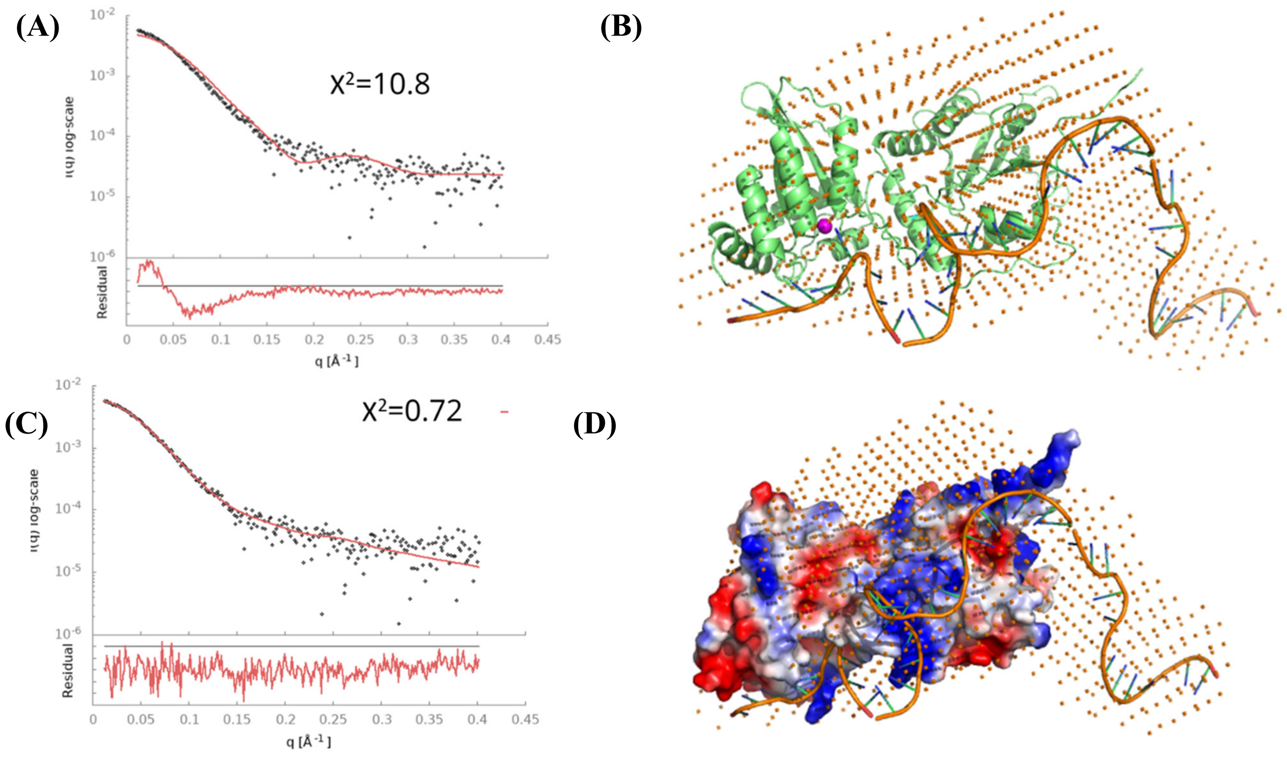

Figure S10. Modelling of A3Gfl/CCdZ-T_{30}-oligo complex.

(**A**) Fit of the monomer from 6P40 without DNA. (**B**) Model of $A3G_{fl}$/ CCdZ-T_{30}-oligo complex (based on 6WMA) and fitted to the SAXS envelope. The modelling was done with PyMol (https://pymol.org/2/) using the homology model with wild type sequence built with YASARA (http://www.yasara.org/) based on the full-length monomer from 6WMA, the catalytically active C-terminal domain of A3G in complex with ssDNA 6BUX[4], and one DNA strand from B-DNA which was broken and combined to fit envelope. (**C**) Fitting of model shown in (**B**) to the SAXS profile. (**D**) The surface of protein coloured according to the charge distribution in complex with CCdZ-T_{30}-oligo.

Specifically, we think that there are multiple modes for DNA interaction. Supplementary Figures S10B and S10D show the alternative model which fit the data only marginally worse than the one in Figure 7. In all of the good fits DNA is interacting with both domains. The fits with no DNA and shorter DNA are much worse. Figure S10A shows the poor fit of the DNA-free monomer to the SAXS data. Moreover, we took the model of Figure 7 and subtracted the final ten 3'-end nucleotides, then the next ten nucleotides, then another ten to leave only a ten-nucleotides bit with dZ bound to the C-terminal. This resulted in gradual worsening of the fit and corresponding increase in chi^2 values. Finally, we constructed a 40-mer model with dZ bound and 30 residues directed away from the protein. This gave a very poor fit to the data (Figure S11):

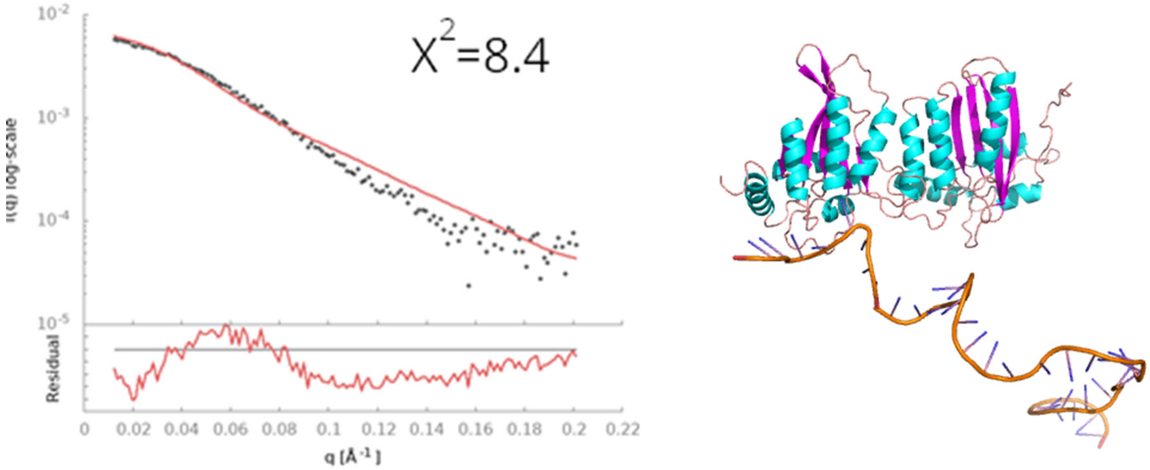

Figure S11. Fit of 40-mer oligonucleotide to data, where only ten residues are in contact in the vicinity of the catalytically active C-terminal domain.

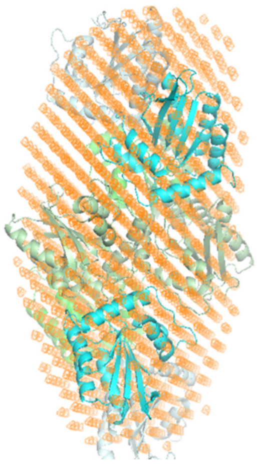

Figure S12. Tetramer model shown in Figure 8 (main text) is superimposed with the A3G$_{fl}$ tetramer envelope model (orange) shown in Figure S4.

References:

1. Brookes, E.; Pérez, J.; Cardinali, B.; Profumo, A.; Vachette, P.; Rocco, M., Fibrinogen species as resolved by HPLC-SAXS data processing within the UltraScan Solution Modeler (US-SOMO) enhanced SAS module. *Journal of Applied Crystallography* **2013,** *46* (6), 1823-1833.
2. Brookes, E.; Rocco, M., Recent advances in the UltraScan SOlution MOdeller (US-SOMO) hydrodynamic and small-angle scattering data analysis and simulation suite. *European Biophysics Journal* **2018,** *47* (7), 855-864.

3. Brookes, E.; Vachette, P.; Rocco, M.; Pérez, J., US-SOMO HPLC-SAXS module: dealing with capillary fouling and extraction of pure component patterns from poorly resolved SEC-SAXS data. *Journal of Applied Crystallography* **2016,** *49* (5), 1827-1841.
4. Franke, D.; Petoukhov, M. V.; Konarev, P. V.; Panjkovich, A.; Tuukkanen, A.; Mertens, H. D. T.; Kikhney, A. G.; Hajizadeh, N. R.; Franklin, J. M.; Jeffries, C. M.; Svergun, D. I., ATSAS 2.8: a comprehensive data analysis suite for small-angle scattering from macromolecular solutions. *J Appl Crystallogr* **2017,** *50* (Pt 4), 1212-1225.
5. Burger, V. M.; Arenas, D. J.; Stultz, C. M., A Structure-free Method for Quantifying Conformational Flexibility in proteins. *Scientific Reports* **2016,** *6* (1), 29040.
6. Svergun, D. I.; Koch, M. H. J., Small-angle scattering studies of biological macromolecules in solution. *Reports on Progress in Physics* **2003,** *66* (10), 1735-1782.
